# A Solar to Chemical Strategy: Green Hydrogen as a Means, Not an End

**DOI:** 10.1002/gch2.202300185

**Published:** 2023-11-20

**Authors:** Gabriel A. A. Diab, Marcos A. R. da Silva, Guilherme F. S. R. Rocha, Luis F. G. Noleto, Andrea Rogolino, João P. de Mesquita, Pablo Jiménez‐Calvo, Ivo F. Teixeira

**Affiliations:** ^1^ Department of Chemistry Federal University of São Carlos Rod. Washington Luís km 235 – SP São Carlos SP 13565‐905 Brazil; ^2^ Cavendish Laboratory University of Cambridge Cambridge CB3 0HE UK; ^3^ Departamento de Química Universidade Federal dos Vales Jequitinhonha e Mucuri Rodovia MGT 367 – Km 583, n° 5000, Alto da Jacuba Diamantina MG 39100 Brazil; ^4^ Department for Materials Sciences Friedrich‐Alexander‐Universität Erlangen‐Nürnberg Martensstrasse 7 D‐91058 Erlangen Germany; ^5^ Chemistry of Thin Film Materials Friedrich‐Alexander‐Universität Erlangen‐Nürnberg IZNF, Cauerstraße 3 D‐91058 Erlangen Germany

**Keywords:** chemical feedstock, green hydrogen, photocatalysis, solar energy, sustainability

## Abstract

Green hydrogen is the key to the chemical industry achieving net zero emissions. The chemical industry is responsible for almost 2% of all CO_2_ emissions, with half of it coming from the production of simple commodity chemicals, such as NH_3_, H_2_O_2_, methanol, and aniline. Despite electrolysis driven by renewable power sources emerging as the most promising way to supply all the green hydrogen required in the production chain of these chemicals, in this review, it is worth noting that the photocatalytic route may be underestimated and can hold a bright future for this topic. In fact, the production of H_2_ by photocatalysis still faces important challenges in terms of activity, engineering, and economic feasibility. However, photocatalytic systems can be tailored to directly convert sunlight and water (or other renewable proton sources) directly into chemicals, enabling a solar‐to‐chemical strategy. Here, a series of recent examples are presented, demonstrating that photocatalysis can be successfully employed to produce the most important commodity chemicals, especially on NH_3_, H_2_O_2_, and chemicals produced by reduction reactions. The replacement of fossil‐derived H_2_ in the synthesis of these chemicals can be disruptive, essentially safeguarding the transition of the chemical industry to a low‐carbon economy.

## Graphical abstract

As the chemical industry seeks to achieve net‐zero emissions, green hydrogen emerges as a critical solution. This work explores the potential of photocatalysis in the production of essential commodity chemicals, such as NH_3_ and H_2_O_2_, driven by sunlight and water. While challenges remain, recent successes demonstrate the viability of this solar‐to‐chemical strategy, presenting a disruptive path toward a low‐carbon economy and the decarbonization of the chemical industry.

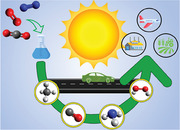

## 1 Introduction

In today's world, addressing the challenges associated with energy production has become vital. Fossil fuel dependence, climate change concerns, geopolitical wars, and the need for sustainable alternatives have propelled the exploration of innovative solutions. Among renewable energies such as wind and hydropower, solar energy stands out as a promising and suitable solution due to the abundant amount of energy available from solar.^[^ ^1^ ^]^

### 1.1 Solar Energy and Low‐Carbon Economy

The abundant and renewable power of the sun provides a direct irradiated energy of 3.8 × 10^24^ J per year.^[^ ^2^ ^]^ Considering the global energy demand was 82,2 × 10^18^ J in 2019, this means that capturing a small portion of the sun's energy could supply such global demand.^[^ ^3^ ^]^ Therefore, solar energy conversion into chemicals offers a way to convert solar photons into storable and transportable fuels or valuable chemicals.^[^ ^4^ ^]^ Nevertheless, the effective capture of solar photons encompasses geographic location, daylight availability, and seasonal variations.^[^ ^5^ ^]^ Solar energy chemical conversion holds the potential to mitigate greenhouse gas emissions, reduce reliance on fossil fuels, and pave the way toward a cleaner and more sustainable energy future.

A low‐carbon economy, in conjunction with a circular sustainable economic system, establishes a series of collective measures, following the Paris Agreement,^[^ ^6^ ^]^ Roadmap and Net Zero by 2050,^[^ ^7^ ^]^ and the Latin American Deep Decarbonization Pathways project.^[^ ^8^ ^]^ One of these measures involves the adoption of energy‐efficient processes, especially for hydrogen (H_2_) production, since is a multifunctional molecule for a wide range of applications, being a key driver for sustainability.^[^ ^9^ ^]^

### 1.2 Hydrogen: Energy Vector and Production

H_2_ has emerged as a crucial element in the pursuit of carbon neutrality. With its versatile applications and potential for zero‐emission energy generation, H_2_ has the capacity to play a key role in achieving carbon neutrality and advancing the transition to renewables.^[^ ^10^ ^]^

The importance of H_2_ as an energy carrier relies upon its high energy density per mass. However, transport considerations need to be taken considering its volumetric density. Regardless of its state (liquid or gas), the energy density of H_2_ per mass is 128 MJ kg^−1^, surpassing the energetic content of conventional fossil fuels such as natural gas, gasoline, propane, and methane by nearly twofold. When compared to ethanol and coal, H_2_ boasts an impressive energy content that is 7 to 8 orders of magnitude higher. However, the volumetric density of H_2_ varies depending on its chemical state. Liquid H_2_ has a density of 71 Kg m^−3^ at −253 °C and 1 bar, while compressed H_2_ gas density is 42 Kg m^−3^ at ambient temperature and 700 bar. In this context, the compression step is vital for practical H_2_ transport, as it allows for higher gas energy carrier quantities in a standard 25 L gas container compared to liquid H_2_. The compression of H_2_ gas varies depending on the applied pressure, ranging from 200 to 700 bar. As a result, the volume unit can accommodate either less or more H_2_ gas content. However, this increased transportability comes at an extra cost due to the added compression step.^[^ ^9^, ^11^ ^]^

Worldwide H_2_ production currently stands at 90 million metric tons, and it is projected to exceed 200 and 500 million metric tons in 2030 and 2050, respectively.^[^ ^12^, ^13^ ^]^ Yet, the majority of production processes rely on fossil fuels (98%), resulting in 900 million metric tons of CO_2_ emissions annually.^[^ ^12^ ^]^ The rising demand is primarily met through energy‐intensive and polluting methods like steam methane reforming (76%) and gasification (22%). In contrast, electrolysis accounts for a minor share of 4%.^[^ ^13^, ^14^ ^]^ Efforts to shift toward cleaner and emerging production methods are needed.

With regard to the process of obtaining and the environmental impact of the production technology, hydrogen is classified as grey, blue, turquoise, white, and green. This ranking ranges from the most environmentally harmful to the most climate‐friendly hydrogen production technologies, including the natural origin.^[^ ^14^ ^]^ Grey hydrogen is currently the most common way to obtain hydrogen. In this process, hydrogen is obtained from steam methane reformation using natural gas and/or methane as precursors. In the reaction, the methane is subjected to high‐temperature and pressure in the presence of steam (H_2_O) and a Ni‐based catalyst to produce H_2_ and CO_2_. In addition to the use of non‐renewable fuel, the process consumes a lot of energy and emits thousands of tons of CO_2_ into the atmosphere. Blue hydrogen is obtained in a similar way to grey hydrogen. However, the greenhouse gases generated are captured and stored, reducing the environmental impact. Although it is estimated that total carbon dioxide equivalent emissions for blue hydrogen are ≈10% lower than for grey hydrogen, which mischaracterizes the technology as being low carbon.^[^ ^15^ ^]^ Turquoise hydrogen is obtained from a different chemical route, the pyrolysis of methane.^[^ ^16^ ^]^ In this process, hydrogen gas and solid carbon are obtained as products. Although not yet used at scale, the strategy can be a less harmful alternative if the thermal process is electrified, using renewable energy sources. White hydrogen is naturally produced deep inside the Earth's crust through a geochemical process. It is a cost‐effective and carbon‐neutral method, comparable to fossil fuels. The production process resembles that of natural gas, including prospecting, site selection, drilling, extraction, and product separation. Environmental protection is of paramount importance in implementing this solution.^[^ ^17^ ^]^ Finally, hydrogen considered “green” is obtained from water splitting using sustainably sustainable sources, such as electricity generated from wind and solar or photocatalysis powered by sunlight.^[^ ^17^ ^]^ Although electrolysis fed by renewable power sources is emerging as the most promising strategy, photocatalysis presents advantages that might be underestimated and could hold a bright future for this topic.

**Figure 1:**
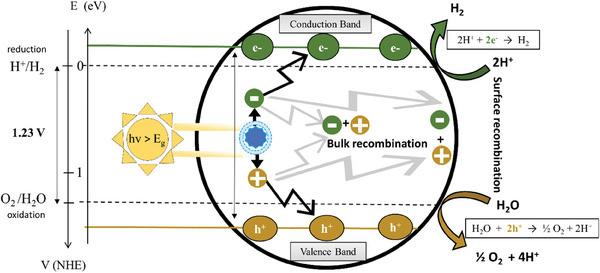
Scheme illustrating the water splitting reaction using a semiconductor‐based photocatalyst. Reproduced with permission.^[^ ^9^ ^]^ Copyright 2023, Elsevier.

The photocatalytic approach can be considered the cleanest, direct, and sustainable method for hydrogen production.^[^ ^18^ ^]^ By mimicking natural photosynthesis, this process utilizes water as its sole substrate. However, unlike the natural counterpart, artificial photocatalysis requires a versatile catalyst and an energy input to initiate the charge transfer and drive the two half‐reactions that effectively split water into oxygen and H_2_ (**Figure** **1**).^[^ ^19^ ^]^

(1) $$H_{2}O+2h^{+}\to \frac{1}{2}O_{2}+2H^{+}$$

(2) $$2H^{+}+2e^{-}\to H_{2}$$

### 1.3 H_2_ Applications in the Chemical Industry

The hydrogen technological roadmap within the chemical industry is quite extensive. Around 90% of the hydrogen produced is consumed in oil refining (hydrocracking, desulfurization) (≈42%), ammonia (≈35%), methanol (≈15%) production, and the remaining ≈8% in other chemicals, such as H_2_O_2_ production and steel manufacturing (direct reduced iron – DRI).^[^ ^20^ ^]^ In 2021, the global demand for hydrogen increased by 5% compared to 2019 (pre‐pandemic), driven mainly by the production of chemicals.^[^ ^21^ ^]^

Currently, ≈70% of the ammonia produced (the main chemical produced from hydrogen) is converted into fertilizer (NPK, urea, and ammonium nitrate) and only 3% of the ammonia is used directly (Ammonia Technology Roadmap). The remainder of global ammonia demand is for a range of industrial applications. Among the main chemical compounds produced from ammonia are pharmaceutical products, amines, acrylonitrile, and nitric acid, which are used, among other applications, in the production of explosives and polymeric materials. Although the main use of urea, a direct derivative of ammonia, obtained from the reaction with carbon dioxide, is as nitrogenous fertilizer, ≈20% of the production is used in different industrial applications, the main one being in the manufacture of resins, especially those based on in urea‐formaldehyde.^[^ ^21^ ^]^ Acrylonitrile is another important derivative of ammonia, obtained from the reaction between propylene, ammonia, and oxygen. Additionally, it is a chemical intermediate for the production of various polymeric materials, including polyacrylonitrile, polyurethanes, and nylons.^[^ ^22^ ^]^

Other hydrogen direct derivatives with significant industrial importance are aniline and hydrogen peroxide. Their direct use and derivatives have major impacts on different industrial sectors, including durable chemical products, paper and cellulose, pharmaceuticals, food, textiles, and others.^[^ ^23^ ^]^ Aniline is obtained from the hydrogenation of nitrobenzene, which is obtained through the reaction of benzene in concentrated nitric acid.^[^ ^24^ ^]^ It is estimated that ≈10 million tons are produced per year for use in the production of polyurethanes from diphenyl methane, 4′‐diisocyanate derivative, rubber additives, and a wide variety of chemical compounds, including pesticides, dyes, and pharmaceuticals.^[^ ^25^ ^]^

Currently, more than 6.5 million metric tons of hydrogen peroxide are produced worldwide, with more than 90% being produced through the anthraquinone AO process, in which hydrogen and oxygen react in the presence of anthraquinone.^[^ ^26^ ^]^ The main demands of hydrogen peroxide are in the pulp and paper industries, as bleaching agents in textiles, liquid and solid detergents, water treatment, semiconductor cleaning, and propylene oxide production.^[^ ^27^ ^]^ In light of the pivotal position role of hydrogen in the chemical industry, developing routes to obtain commodity chemicals, such as ammonia, aniline, and hydrogen peroxide, without relying on fossil‐derived hydrogen is of paramount importance to enable a transition to a sustainable and CO_2_‐free chemical industry.

### 1.4 Advantages of H_2_ Direct Use

Photocatalysis as well as electrolysis can provide a route to green hydrogen production. However, the photocatalysis approach holds much greater potential; it can be employed as a strategy to directly convert the hydrogen generated from water into chemicals. The direct utilization of H_2_ for chemical production offers environmental, renewable energy, versatility, energy efficiency, and sustainability advantages, making it an attractive option for the chemical industry.^[^ ^28^ ^]^

In this direction, using H_2_ directly to produce chemicals offers several advantages:

- Environmental Benefits: Chemical production can significantly reduce carbon emissions compared to conventional methods. By avoiding intermediate steps or fossil fuel inputs, the process can help mitigate greenhouse gas emissions and contribute to a more sustainable and low‐carbon economy.
- Renewable and Clean Energy Source: Utilizing this direct conversion of green H_2_ for chemical strategy leads to greener and more sustainable processes, where ideally all the energy input will come from sunlight.
- Versatile Feedstock: As mentioned, H_2_ is a platform molecule in the chemical industry, participating in various reactions enabling the production of a wide range of chemicals. Its versatility allows for the synthesis of different compounds and can serve as a valuable building block in various industrial processes.
- Energy Efficiency: Green H_2_ generated by photocatalysis often exhibits high selectivity and can operate under milder conditions, reducing energy requirements compared to traditional processes. With efficiency gains, this might translate into cost savings and reduced resource consumption in the future.
- Reduced Dependency on Fossil Fuels: By utilizing green H_2_ directly, the reliance on fossil fuel feedstocks can be minimized. This reduces vulnerability to fossil fuel price fluctuations and supply disruptions, creating a more stable and sustainable chemical industry. In other words, the chemical industry relies mainly on the widely available sunlight and not on fossil fuels.
- Potential for Circular Economy: The H_2_ generated by photocatalysis can be helped by sacrificial reagents, which can be chosen according to the principles of a circular economy. It enables the conversion of waste or by‐products into hydrogen sources and ultimately into valuable chemicals. This approach promotes resource efficiency and waste reduction, contributing to a more circular and sustainable industrial ecosystem.

Therefore, integrating the production of H_2_ and chemical conversion processes within the same site offers significant advantages. By eliminating the need for separate storage facilities, a continuous process can be achieved, allowing ultimately energy storage into chemicals. This strategy solves one of the greatest challenges in the hydrogen economy, which is H_2_ storage. This integration has the potential to optimize resource utilization, reduce costs, and enhance the overall efficiency of the hydrogen‐based chemical production system.

## 2 Photocatalytic‐driven Ammonia Production

Currently, there is great interest from the scientific community worldwide in developing technological alternatives for the synthesis of ammonia (or atmospheric nitrogen fixation) to replace the Haber‐Bosch process, which is widely regarded as costly from both environmental and energy standpoints.^[^ ^29^ ^]^

Ammonia is the second most produced chemical in the world, with an estimated production of ≈200 million tons per year.^[^ ^30^ ^]^ However, its significance extends beyond sheer production volume, as it is considered one of the most crucial chemicals due to its immense demand in food production (primarily as fertilizers) and its widespread usage in the chemical industry. Furthermore, ammonia has the potential to play a central role as a hydrogen storage strategy within the future hydrogen economy. Regarding hydrogen storage, in addition to an H_2_ content of 17.65%, NH_3_ has been recommended as the most technically viable option because of its properties that allow liquefaction under milder temperature and pressure conditions. Additionally, it offers greater safety for storage and transport due to its low vapor pressure and higher boiling point.^[^ ^31^ ^]^

Worldwide ammonia production is dominated by the Haber–Bosch process. In this process, gaseous nitrogen (N_2_) and gaseous hydrogen (H_2_) react at elevated temperature (400‐°C) and pressure (10–15 MPa), over an iron (Fe)‐based catalyst. In addition to the extreme reaction conditions, the process consumes ≈1‐% of the world's energy production^[^ ^32^ ^]^ and contributes 1.2% of the total global CO_2_ emission, as hydrogen gas is obtained using non‐renewable feedstock, specifically steam methane reforming (CH_4_ + 2H_2_O → 4H_2_ + CO_2_) or coal gasification (C + 2H_2_O → 2H_2_ + CO_2_). Despite the high energy and environmental cost, the use of the Haber‐Bosch process within the context of an economy based on the use of fossil fuels had a positive impact on the economy and food production.^[^ ^33^ ^]^ However, in the process of global decarbonization, it is urgent to change the ammonia production process as a way to drastically reduce both CO_2_ emissions and high energy consumption. As it was at the beginning of the last century, ammonia synthesis is considered a great and fascinating challenge of the 21st century, playing a key role in a sustainable future.^[^ ^34^ ^]^

The electrification of the Haber‐Bosch process is a more realistic alternative at first, as it replaces the use of fossil sources by electrolysis of water to supply hydrogen.^[^ ^35^ ^]^ On the other hand, the intrinsic high‐energy demand of the process remains. Evidently, the use of alternative sources of energy generation can justify this strategy, but from a future perspective, the industrial process will need significant changes for the desired energy revolution to be achieved. This realization will only happen by exploring green, sustainable, mild, and economic nitrogen fixation strategies.^[^ ^36^ ^]^

**Figure 2:**
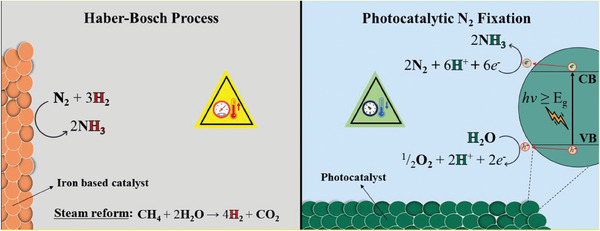
Comparative scheme representing the ammonia production from the traditional Haber‐Bosch process and the photocatalytic approach where water is the electron donor.

In this context, the nitrogen fix employs a catalyst directly photoactivated by sunlight to promote the reduction of atmospheric N_2_ into NH_3_, in which water or other sustainable proton source is the source of protons, under ambient conditions. This is the strategy that holds the greatest potential for enabling green and sustainable ammonia production since it ideally does not require the production of hydrogen gas and the use of high temperature and pressure (**Figure** **2**).

In the Haber‐Bosch process, molecular bonds are broken to form nitrogen and hydrogen atoms through a dissociative mechanism,^[^ ^37^ ^]^ as adding the first hydrogen atom is challenging (Equation 3).^[^ ^38^ ^]^ In nitrogen fixation using photocatalysis, molecular nitrogen adsorbed on the catalyst surface is reduced in the conduction band by excited electrons from the valence band (Equation 4), and the electronic structure is restored through the oxidation of water into molecular oxygen (Equation 5). This oxidation provides the protons for the formation of NH_3_.^[^ ^39^ ^]^ This uphill reaction requires a potential greater than 1.14 eV.

(3) $$N_{2(g)}+H_{\left(\mathrm{aq}\right)}^{+}+e^{-}\to N_{2}H_{\left(\mathrm{ads}\right)}\left(-3.2\;V\;\mathrm{vs}\;\mathrm{RHE}\right)$$

(4) $$N_{2(g)}+6H_{\left(\mathrm{aq}\right)}^{+}+6e^{-}\to 2NH_{3(g)}\left(0.092\;\;V\;\mathrm{vs}\;\mathrm{SHE}\right)$$

(5) $$H_{2}O_{\left(l\right)}\to \frac{1}{2}O_{2(g)}+2H_{\left(\mathrm{aq}\right)}^{+}+2e^{-}\left(1.23\;\;V\;\mathrm{vs}\;\mathrm{SHE}\right)$$

The first demonstration of nitrogen fixation through photocatalysis was reported in 1977 by Schrauzer and Guth.^[^ ^40^ ^]^ Subsequently, Schrauzer et al. showed that N_2_ photofixation occurs on finely dispersed titanium minerals, such as rutile, present on sand surfaces from different geographic locations.^[^ ^41^ ^]^ From this pioneering demonstrative studies on the possibility of producing ammonia on the surface of a catalyst using light have given rise to a new research sector within photocatalysis, which has seen significant growth in the last decade and even greater interest from researchers in recent years. The growing interest is evident from the results generated by the ISI Web of Science database using “nitrogen fixation photocatalysis”, “ammonia photocatalysis” or “nitrogen photoreduction nitrogen fixation”. Between 2002 and 2012, 293 studies were reported. From 2013 to 2023, out of 1317 works, 793 were published from 2020 onward, demonstrating extensive recent research interest.

**Figure 3:**
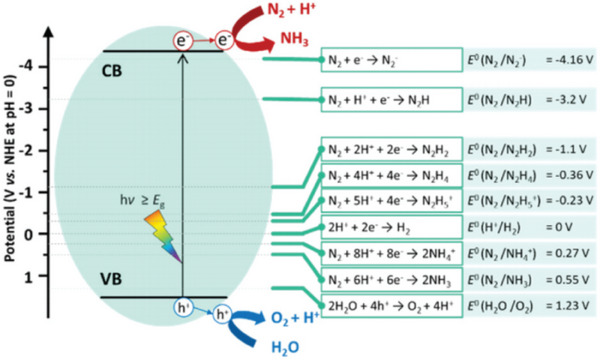
Schematic representation of photocatalytic N_2_ reduction on a photoactivated semiconductor and the respective redox potentials associated with the reduction of N_2_ from 1 to 6 electrons. For comparison purposes, the redox potentials of the water are also shown. Reproduced with permission.^[^ ^42^ ^]^ Copyright 2021, Royal Society of Chemistry.

To facilitate the discussion of the main challenges associated with the application of photocatalysis for the production of ammonia, it is necessary to understand the basic mechanism of photocatalysis. First, we will consider that the photocatalyst has efficient photon absorption and the valence and conduction bands are ideally positioned for the oxidation and reduction of the target molecules, in this case N_2_ and an electron donor, such as water or another sacrificial agent. After the absorption of photons with energy equivalent to the band gap of the semiconductor, the electron donor is oxidized in the valence band, while N_2_ molecules are adsorbed on the surface and reduced in the conduction band by the photoexcited electrons. **Figure** **3** shows the redox potentials of the reduction of the N_2_ molecule (1 electron) on the surface of a photoactivated semiconductor.

Currently, solar‐driven nitrogen fixation faces several main challenges, including the inhibition of h^+^/e^−^ recombination, N_2_ chemisorption, and activation.^[^ ^43^ ^]^ As a consequence, the production rates for most studied photocatalysts remain significantly lower than desired, making the success of the solar fixation of N_2_ still distant.^[^ ^39^ ^]^ In fact, the vast majority of results show production on the order of µmol h^−1^ g^−1^.^[^ ^44^ ^]^ Despite the photocatalytic ammonia production using water as an electron donor being the most attractive and sustainable condition, the kinetics of the process represents a challenge due to the complexity involving the multi‐electron oxidation of H_2_O, which is inhibited by the rapid recombination rate of photogenerated electron‐hole pairs. Introducing sacrificial agents of renewable origins, such as methanol, glycerol, or even wastewater,^[^ ^45^ ^]^ and biomass^[^ ^46^ ^]^ could improve charge separation and consequently the overall quantum efficiency of the reaction, as sacrificial electron donors supply the holes in the semiconductor the presence of sacrificial agents may also inhibit the oxidation of the ammonia produced,^[^ ^47^ ^]^ capturing eventual oxidizing radicals formed in parallel reactions. A more interesting alternative to the use of sacrificial agents lies in coupling the ammonia photofixation with the conversion of sacrificial agents through selective oxidation to value‐added chemicals.^[^ ^48^ ^]^

To date, TiO_2_ is one of the most investigated photocatalysts for this reaction due to its superior photoactivity, but its high band gap (≈3.2 eV) and poor utilization of the solar spectrum have motivated the application and development of new materials. This has led to several modification strategies over recent years, such as noble metal deposition (co‐catalysis),^[^ ^49^ ^]^ defect engineering,^[^ ^50^ ^]^ doping, and surface tailoring. For example, Ding et al. found that N‐doping in Ti_3_C_2_‐TiO_2_ improved the ammonia production yield to 415.6 µmol h^−1^ g^−1^ (8 times that of pure TiO_2_) due to the enhanced charge separation facilitated by the doping.^[^ ^51^ ^]^ Li et al. also demonstrated an increase from 31.50 to 80 µmol h^−1^ g^−1^ using N‐doping in TiO_2_.^[^ ^52^ ^]^ This strategy often generates defects and oxygen vacancies that promote better adsorption and activation of N_2_, as well as increased separation and migration of photogenerated carriers. TiO_2_ loaded with Sr (Sr/TiO_2_) also exhibited efficient separation and migration of photogenerated charges. This catalyst yielded an NH_3_ rate of 32.2 µmol h^−1^ g^−1^ (547.4 mg h^−1^ g^−1^) under full spectrum light and 20.8 µmol h^−1^ g^−1^ (353.9 mg h^−1^ g^−1^) under visible light illumination, surpassing Sb nanosheets and pure a‐TiO_2_ by factors of 2 and 16, respectively.^[^ ^53^ ^]^

Various materials have been considered for photocatalytic nitrogen fixation (**Table** **1**), such as metal oxides, metal sulfides, layered double hydroxides, and carbon‐based materials including carbon nitride,^[^ ^54^, ^55^ ^]^ bismuth‐based materials,^[^ ^56^ ^]^ single atom catalysis,^[^ ^55^, ^57^, ^58^ ^]^ and metal‐organic frameworks (MOFs).^[^ ^59^ ^]^ Among these materials, the most promising results for photocatalytic nitrogen fixation have been observed for bismuth‐based materials. In 2015, Li et al. reported one of the first studies on catalysts based on bismuth oxyhalides.^[^ ^60^ ^]^ The authors prepared an active catalyst in the visible region absorption based on BiOBr nanosheets containing oxygen vacancies on the exposed 001 facets. The visible‐light‐driven photocatalytic N_2_ fixation rate was estimated to be 104.2 µmol h^−1^ g^−1^ when using water as a proton donor, without the need for precious‐metal cocatalysts. More recently, Li et al. presented Bi_5_O_7_Br as a photocatalyst with well‐defined nanotube structures under visible‐light irradiation, achieving an impressive rate of 12.72 mM g^−1^ h^−1^ for nitrogen fixation.^[^ ^61^ ^]^

**Table 1:** Summary of different photocatalysts applied for N_2_ fixation.

Catalyst	Hole scavenger employed	Catalyst mass [mg]	Light wavelength [nm	NH_3_ rate [µmol g^−1^ h^−1^]	AQY [%]	Reference
Ti_3_C_2_‐TiO_2_	CH_3_OH (20%)	50	420	415.6	N/A	[51]
NTO.5	H_2_O	N/A	350–780	80.09	0.07 (375 nm)	[52]
Sb/TiO_2_	MeOH (20%)	N/A	>420	20.82	N/A	[53]
BOB‐OV	H_2_O	50	420–780	104.2	0.23 (420 nm)^a)^	[60]
Bi_5_O_7_Br0	H_2_O	25	400	12 720	N/A	[61]
Ru‐CCN.05	H_2_O	50	Xenon lamp	132,69	N/A	[58]
Cu‐CN	EtOH (20%)	50	420	186	N/A	[65]
Fe‐T‐S	H_2_O	50	Xenon lamp	32	N/A	[66]

^a)^ External quantum efficiency (EQE).

Unlike heterogeneous catalysts, single‐atom catalysts (SACs) in general offer the high efficiency and selectivity presented by homogeneous catalysts, but with the robustness and manipulation advantages associated with heterogeneous catalysis.^[^ ^62^ ^]^ SACs represent the new frontier in the development of photocatalysts,^[^ ^63^ ^]^ bringing several important concepts. Apart from their high atomic efficiency, SACs possess a unique electronic structure and adjustable coordination environments for the active sites,^[^ ^64^ ^]^ which have been successfully exploited in the photocatalytic nitrogen fixation reaction. Li et al. prepared a single‐atom ruthenium catalyst on the surface of the graphitic carbon nitride (g‐C_3_N_4_). They obtained a material with 5% ruthenium and significant structural defects (cyano groups) on the surface of the support, which was then for nitrogen fixation using only water as a proton source. The catalytic performance of the material containing single atom sites was better than that observed for bare g‐C_3_N_4_ by 1,5 times. The rate of photocatalytic nitrogen fixation obtained in ultra‐pure water was 132 µmol g^−1^ h^−1^.^[^ ^58^ ^]^

Xie et al. reported a single Cu atom supported on porous g‐C_3_N_4_. The authors demonstrated that Cu single atoms were immobilized at the defect sites of g‐C_3_N_4_ via the formation of Cu‐N bonds. By integrating nitrogen adsorption sites and the electron accumulation on the Cu single atom, the catalytic activity of SAC was nine times higher than that observed for pristine g‐C_3_N_4_, achieving an ammonia yield of 186 µmol g^−1^ h^−1^.^[^ ^65^ ^]^ Similarly, Fe single atom immobilized on mesoporous TiO_2_‐SiO_2_ displayed an ammonia generation rate of 32 µmol g^–1^ h^‐^ without any sacrificial agent. Experimental and theoretical calculations confirmed the formation of photoinduced high‐valent Fe(IV) species, which played a dual role in water oxidation and N_2_ hydrogenation, primarily at neighboring oxygen vacancies.^[^ ^66^ ^]^

In fact, overcoming the inhibition of recombination and increasing exciton lifetime are crucial challenges for photocatalysis in sustainable ammonia synthesis. Developing heterojunctions represents an interesting and rational strategy to address these issues.^[^ ^34^ ^]^ Although some works show positive results, type I heterostructures generally yield lower results compared to type II heterojunctions. This difference arises because, in type I heterostructures, electrons and holes are concentrated within the same semiconductor, theoretically facilitating recombination. In contrast, in type II heterostructures, while the holes are concentrated on semiconductor A, the excited electrons are concentrated on semiconductor B. This spatial separation delays recombination, increasing the likelihood of electron transfer reaction on the photocatalyst's surface.

Defect engineering, cocatalysis, and heterojunctions strategies have indeed led to improvements in the activity, but they have generally fallen short of meeting established objectives. Nevertheless, there has been a significant surge in interest in this field over the past 5 years, which is expected to yield more promising results in a relatively short period. Many of the primary challenges have already been identified and addressed.^[^ ^67^ ^]^ Due to the slow oxidation rate of water as a proton supplier for nitrogen reduction, the use of sacrificial agents appears to be inevitable. In this context, the coupling of an ammonia production system with industrial waste treatment or the synthesis of valuable by‐products should be considered an intriguing strategy for the feasibility of photocatalytic nitrogen fixation. However, the development of these coupled systems presents challenges similar in magnitude to ammonia photofixation itself. This requires the development of specific catalysts with unique structural and electronic characteristics for each photoreactor, underscoring the pivotal role of photoreactor design in this process.

## 3 Photocatalytic‐driven Hydrogen Peroxide Production

Hydrogen peroxide is one of the simplest molecules in nature with enormous relevance to industry. Its varied applications ranging from the biomedical industry to cosmetics, establish it as one of the most versatile light chemicals. It plays an essential role in the paper industry as a bleaching agent for cellulose,^[^ ^68^ ^]^ it is a powerful bactericidal agent extensively used in water treatment,^[^ ^69^ ^]^ where it also contributes to the degradation of organic pollutants.^[^ ^70^ ^]^ More importantly, because of its chemical reactivity, it can be considered a green oxidant, only releasing water and oxygen as by‐products, which is highly relevant to the pharmaceutical industry.^[^ ^27^ ^]^ Hydrogen peroxide also finds a useful generator of reactive oxygen species (ROS) in the iron‐catalyzed Fenton reaction.^[^ ^71^ ^]^ Lastly, it is a promising substrate for fuel cells, where it can play a dual role, contributing to current generation at both the cathode and the anode, enabling electricity generation from a single chemical source.^[^ ^72^ ^]^ The global market value of hydrogen peroxide was estimated to be ≈3.2 billion US dollars in 2022 and is expected to increase to 4.0 billion in the next five years.^[^ ^73^ ^]^

Hydrogen peroxide is a valuable chemical that can be synthesized from the second most abundant gas in our atmosphere (i.e., O_2_). Three main synthetic routes can be identified: i) redox neutral comproportionation of H_2_ and O_2_, ii) reductive oxygen reduction reaction (ORR) reaction, and iii) oxidative water oxidation reaction (WOR). Although an excellent example of atom economy,^[^ ^74^ ^]^ it is evident that the first method must rely on green hydrogen for sustainable implementation. Nevertheless, hydrogen/oxygen mixtures pose risks of explosion, as well as engineering challenges related to gas storage and delivery.^[^ ^75^ ^]^ Therefore, redox conversion from abundant O_2_ or H_2_O is the preferred strategy for sustainable H_2_O_2_ synthesis. Such chemical reactions easily occur at mild conditions in the regime of electrocatalysis, where electricity can be generated from wind turbines, photovoltaic cells, or through photocatalysis. The latter is usually fulfilled by means of light‐absorbing semiconductors, that in turn, transfer excited electrons or holes to adsorbed substrates. Catalyst design for direct solar‐driven production of hydrogen peroxide requires a thorough knowledge of the main redox couples involving O_2_, H_2_O, and H_2_O_2_ in aqueous solution:

(6) $$O_{2(g)}+4H_{\left(\mathrm{aq}\right)}^{+}+4e^{-}\to 2H_{2}O\left(1.23\;V\;\mathrm{vs}\;\mathrm{SHE}\right)$$

(7) $$O_{2(g)}+2H_{\left(\mathrm{aq}\right)}^{+}+2e^{-}\to H_{2}O_{2}\left(0.68\;V\;\mathrm{vs}\;\mathrm{SHE}\right)$$

(8) $$O_{2(g)}+H_{\left(\mathrm{aq}\right)}^{+}+e^{-}\to {\mathrm{HOO}}^{•}\left(-0.13\;V\;\mathrm{vs}\;\mathrm{SHE}\right)$$

(9) $$H_{2}O_{2(\mathrm{aq})}+2H_{\left(\mathrm{aq}\right)}^{+}+2e^{-}\to 2H_{2}O\;\left(1.77\;V\;\mathrm{vs}\;\mathrm{SHE}\right)$$

(10) $$2H^{+}+2e^{-}\to H_{2(g)}\;\left(0.00\;V\;\mathrm{vs}\;\mathrm{SHE}\right)$$

It is important to note that all these reactions are highly pH‐dependent. Therefore, the redox potential must be appropriately calculated depending on the reaction conditions. Indeed, the thermodynamic potentials can vary by several hundred millivolts between acidic and alkaline environments. For example, the 2e^−^ ORR (Equation 7) to H_2_O_2_ is significantly less spontaneous at pH 7, with a redox potential as low as 0.27 V. Although it is common practice to evaluate the activity and selectivity of a semiconductor photocatalyst by comparing the position of its conduction and valence bands to reduction potentials, these are sometimes erroneously taken at their reference state, that is at an impractical pH = 0.

**Figure 4:**
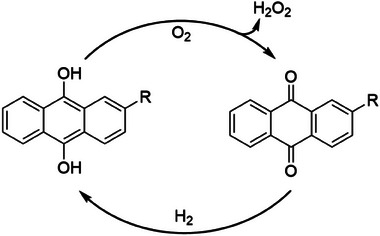
The industrial anthraquinone process.

Hydrogen peroxide was first generated by electrolysis, but since the 1940s, it has been produced on a large scale through the so‐called “anthraquinone process” (**Figure** **4**). In this method, a 2‐alkyl anthraquinone is first reduced to the corresponding hydroquinone using hydrogen gas over a Pt catalyst. Hydroquinone is then oxidized back into air, and as a result, O_2_ is reduced to H_2_O_2_, and the cycle is repeated.^[^ ^76^, ^77^ ^]^ Despite its high yield, the process has several drawbacks: i) the use of pressurized H_2_, ii) the need for a noble catalytic metal, iii) the generation of by‐products from parasitic anthraquinone ring hydrogenation, and iv) the need for a liquid‐liquid extraction step to isolate H_2_O_2_ from the organic phase. Such limitations urged scientists to find innovative and sustainable electrocatalytic or direct photocatalytic methods for H_2_O_2_ generation.^[^ ^78^ ^]^ Furthermore, it is worth highlighting that the H_2_ input commonly used in the chemical industry is fossil‐derived H_2_ (grey hydrogen).

**Figure 5:**
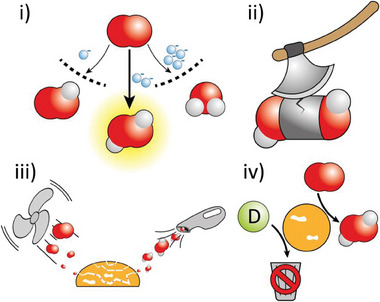
Challenges to be addressed for developments in sustainable H_2_O_2_ synthesis. i) Enhanced selectivity toward 2e^−^ ORR to H_2_O_2_; ii) Suppression of H_2_O_2_ decomposition; iii) Improved mass transfer of reactants and products; iv) Alternative solutions to sacrificial electron donors.

In the context of light‐driven synthesis, many catalytic materials have been tested, including metal oxides, metal‐organic frameworks, and carbon nitrides.^[^ ^79^ ^]^ Photocatalytic production of H_2_O_2_ has been almost entirely concerned with ORR. While H_2_O_2_ can be successfully synthesized in good yields using a variety of materials, several challenges still need to be addressed for a viable large‐scale alternative. Some of the issues to be tackled are common to all photocatalysts: light‐absorption should be enhanced in order to utilize a larger portion of sunlight, charge separation efficiency should be increased, and charge recombination should be suppressed as much as possible.^[^ ^80^ ^]^ However, challenges more specifically associated with H_2_O_2_ synthesis can be identified (**Figure** **5**):

- Selectivity of ORR should be increased: Oxygen can be either reduced through a 4e^−^ (Equation 6), 2e^−^ (Equation 7), or 1e^−^ (Equation 8) route, yielding H_2_O, H_2_O_2_ and HOO·, respectively. Trade‐offs between thermodynamics and kinetics can be identified. The first reaction is thermodynamically favored, but it requires a kinetically challenging 4‐electron transfer. On the other hand, the generation of hydroperoxyl radicals has a much lower reduction potential, but it can rapidly occur by a single electron transfer. Moreover, for a reductive enough photocatalyst, the 1e^−^ route will dominate in most scenarios. Nominally, for ideal ORR selectivity toward H_2_O_2_, the conduction band of a photocatalytic semiconductor (or, equivalently, the lowest unoccupied molecular orbital of a molecular catalyst) should lie between the reduction potential of (Equation 7) and (Equation 8), that is between −0.54 V and 0.27 V versus SHE at pH 7, or −0.13 V and 0.68 V at pH 0. As illustrated in the following section, selectivity can also be shifted with different strategies, for example working on the morphology of the catalyst to favor adsorption of transient species.^[^ ^81^ ^]^
- Decomposition of synthesized H_2_O_2_ should be suppressed: Hydrogen peroxide is susceptible to disproportionation to H_2_O and O_2_ even at mild conditions. This reaction can even be accelerated in the presence of transition metals. In the design of metal‐based catalysts for ORR, it is crucial to ensure that synthesized hydrogen peroxide is not decomposed in‐situ on the same material. For example, Ag is well‐known to catalyze H_2_O_2_ disproportionation.^[^ ^82^ ^]^ More studies suggest that Pt and Pd lead to even faster decomposition rates, followed by Au.^[^ ^83^ ^]^ In addition to the material, the illumination source also plays a major role. UV light is known to cleave the peroxide bond.^[^ ^84^ ^]^ Consequently, the use of band‐pass filters to avoid shorter wavelengths is highly recommended. For the same reason, systems relying on TiO_2_ excitation should be evaluated with care, since a remarkable conversion of O_2_ might still result in low H_2_O_2_ yields because of its UV‐induced degradation to radicals.
- Mass transfer of O_2_ should be improved: In an aqueous solution, the extent of ORR is dictated by the liquid‐air contact area and the concentration of dissolved gas. Oxygen is usually bubbled to increase the reaction yield, but once the dissolved gas is consumed, mass transfer may become the limiting step of the reaction. It should be remembered that oxygen solubility in water at room temperature and 1 atm is ≈1.22 mmol L^−1^.^[^ ^85^ ^]^ Strategies to overcome this issue might involve multiphasic systems or gas diffusion layers.
- The reaction ideally should occur without any sacrificial electron donor: This last challenge will probably be the hardest to address. Indeed, most reports on photocatalytic H_2_O_2_ synthesis rely on alcohols or amines as sacrificial electron donors. Smarter electron‐donating substrates might be chosen, in the view of a circular economy. For example, glycerol waste from biofuel synthesis^[^ ^86^ ^]^ or ethylene glycol from PET recycling are valid reducing substrates.^[^ ^87^ ^]^ In an ideal scenario, H_2_O_2_ should be produced from the convergence of 2e^−^ ORR and WOR (comproportionation). However, the latter is a surprisingly thermodynamically challenging reaction, often replaced by 4e^−^ oxidation to O_2_, with an admittedly very sluggish kinetics. 2e^−^ WOR has been scarcely reported, mainly in electrochemical devices,^[^ ^88^ ^]^ and therefore deserves more consideration.

In the following paragraph, possible approaches to each of these challenges will be provided with reference to the state‐of‐the‐art.

### 3.1 Approaches to Light‐Driven H_2_O_2_ Production

Selective synthesis of hydrogen peroxide is commonly achieved through careful band engineering, particularly for the ORR scenario, the conduction band should not lie at too negative potentials to prevent the formation of HOO· species. In general terms, band engineering is achieved by hybridization of the native catalyst with a variety of materials. We have summarized the most relevant photocatalyst employed for H_2_O_2_ production through both WOR and ORR pathways (**Table** **2**).

**Table 2:** Summary of different photocatalysts applied for H_2_O_2_ production.

Catalyst	Mechanism	Catalyst mass [mg]	Light wavelength [nm]	H_2_O_2_ rate [µmol g^−1^ h^−1^]	AQY [%]	Ref
CDs@CTFs	ORR/WOR	30	Sunlight simulator	≈633.3	0.93% (505 nm)	[80]
g‐C_3_N_4_	ORR	20	420–500	125	0.07 (375 nm)	[81]
Au/WO_3_	ORR	50	420	18.7	N/A	[83]
PCN‐NaCA	ORR	10	Sunlight simulator	1870	27.6 (380 nm)	[86]
g‐C_3_N_4_‐CNTs	ORR/WOR	50	420–700	658	N/A	[90]
Au_0.2_/BiVO_4_	ORR	50	>420	2412	0.24 (420 nm)	[93]
H‐PHI	ORR	5	410	1556	0.86	[95]
MIL‐R7	ORR	5	>420	≈1073	N/A	[96]
TiO_2_	ORR	30	300 or 420	719.5 (300 nm)	N/A	[102]
Sb‐SAPC15	ORR/WOR	100	400–500	91 149	17.6 (420 nm)	[103]
Co_1_/AQ/C_3_N_4_	ORR	6	Xenon lamp	57.5	0.054%^a)^	[105]
TiO_2_	ORR	50	280–400	335	29.1% (334 nm) 0.5 (Xe lamp)	[106]
g‐C_3_N_4_/PDI‐BN_0.2_‐rGO_0.05_	WOR	50	420–500	74	7.3 % (420 nm)	[107]

^a)^ Full spectrum.

**Figure 6:**
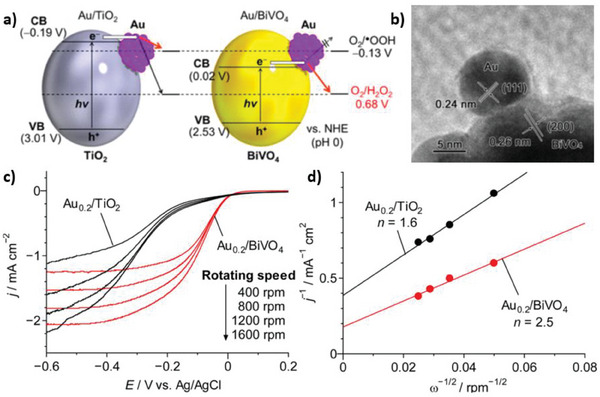
a) Illustration of Au/TiO_2_ and Au/BiVO_4_ photocatalysts with relevant band energies and reduction potentials. b) High‐resolution transmission electron microscopy (HR‐TEM) of Au/BiVO_4_. c,d) Koutecky‐Levich analysis of two ORR photocatalysts. c) Linear‐sweep voltammograms measured on a rotating disk electrode at different rotating speeds. d) Koutecky‐Levich plots at the constant potential of −0.3 V versus Ag/AgCl. Reproduced with permission.^[^ ^93^ ^]^ Copyright 2016, American Chemical Society.

Covalent functionalization has been extensively investigated for graphitic carbon nitrides.^[^ ^89^ ^]^ While these materials are safe, inexpensive, and responsive to visible light, they are non‐selective and not exceptionally active due to charge recombination. Protocols are available for the covalent attachment of carbon nitrides to carbon‐rich materials like carbon nanotubes,^[^ ^90^ ^]^ or charge‐separating centers like polyoxometalates.^[^ ^91^ ^]^ On the other hand, BiVO_4_ has intrinsically ideal band positions for the selective 2e^−^ ORR, with its conduction band approximately located at 0.02 V versus SHE.^[^ ^92^ ^]^ It is inexpensive and possesses a low bandgap (≈ 2.5 eV) compatible with the absorption of visible light. Nevertheless, it needs catalytic materials to improve chemical conversion. The most viable catalytic materials are noble metal nanoparticles, as was demonstrated with Au (**Figure** **6**a,b).^[^ ^93^ ^]^

Nevertheless, certain photocatalysts can shift the ORR selectivity toward H_2_O_2_ by lowering the adsorption energy of key intermediates. This surface chemistry effect was first demonstrated for carbon nitrides in Shiraishi's group which detected a 1,4‐endoperoxide species on the surface of carbon nitrides by means of Raman spectroscopy.^[^ ^81^ ^]^ The full mechanism is described in detail in the following paragraph.

In H_2_O_2_ synthesis, selectivity is often overlooked, and the performance of a photocatalytic method is primarily evaluated in terms of the yield or rate of hydrogen peroxide production. However, from this approach, many photocatalytic materials might be discarded because deemed as inactive, while in fact, they could only lack selectivity. To resolve this uncertainty, it is highly recommended that authors consider an electrochemical Koutecky‐Levich analysis.^[^ ^94^ ^]^ This analysis is performed on a rotating disk electrode (RDE) and can easily provide information on the average number of electrons transferred from the catalyst to the substrate, with fast experimental procedures and minor data analysis required, provided that the catalyst is successfully deposited on the working electrode. The technique consists of recording linear‐sweep voltammetry data in an RDE at different rotating speeds. The inverse of the current density at a constant voltage is then plotted against the inverse of the square root of the rotating speed and fitted by linear regression. The number of electrons transferred in the redox process can be determined by the slope of such a plot, using the following relationships:

(11) $$j^{-1}=k+B^{-1}\omega^{-\frac{1}{2}}$$

(12) $$B=0.2nFv^{-\frac{1}{6}}CD^{\frac{2}{3}}$$

where *k* is a constant, *j* is the current density, w is the rotating speed, *n* is the average number of transferred electrons, *F* is the Faraday constant, n is the kinetic viscosity of water (0.01 cm^2^ s^−1^), *C* is the concentration of O_2_ in solution and *D* is the diffusion coefficient of O_2_. A Koutecky‐Levich analysis was performed for the Au/BiVO_4_ photocatalyst and compared to Au/TiO_2_ (Figure 6c,d).^[^ ^93^ ^]^ The slopes of the j^−1^‐ω^−1/2^ curves for the two catalysts at a relevant potential resulted in *n* values of 2.5 and 1.6, respectively. The result indicated that Au/BiVO_4_ has a role in promoting the 2e^−^ ORR, while with Au/TiO_2_ the 1e^−^ pathway dominates.

Once H_2_O_2_ is selectivity produced, care should be taken to prevent its decomposition due to its very spontaneous disproportionation. Even though H_2_O_2_ is synthesized with remarkable selectivity, final yields might be low due to *in‐situ* degradation on the catalyst. For example, poly (heptazine imides) (PHI) single‐atom photocatalysts comprising a variety of noble and abundant transition metals were recently tested for photocatalytic ORR. Surprisingly, the best activity was achieved with the supposedly non‐catalytic Na‐PHI precursor.^[^ ^95^ ^]^ Indeed, it was observed that when fresh H_2_O_2_ was mixed with a transition‐metal single‐atom catalyst – namely Fe‐PHI – it was consumed faster than on Na‐PHI, with the rate of decomposition increasing with the metal loading. Noble metals are particularly problematic catalysts. In particular, Pt, Pd, and Ag should be avoided, because of their demonstrated high rate of H_2_O_2_ decomposition.

**Figure 7:**
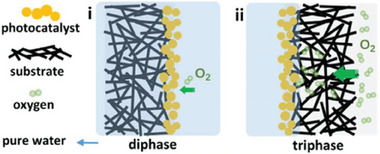
Schematic illustration of i) a biphasic and ii) a triphasic reactor for catalytic H_2_O_2_ synthesis. Reproduced with permission.^[^ ^98^ ^]^ Copyright 2021, John Wiley and Sons.

To address this issue, alternative strategies can be adopted to facilitate the fast and efficient desorption of the product from the catalyst. This was demonstrated in a biphasic system, where a hydrophobic photocatalyst was dispersed in an organic phase, while hydrogen peroxide was concentrated in water.^[^ ^96^ ^]^ This strategy also mimics the industrial anthraquinone process, where H_2_O_2_ is extracted in an aqueous solution after reaction in the organic phase. Triphasic systems might particularly improve the reaction since they address both product desorption and enhanced gaseous reactant mass transfer. Instead of organic solvents, oxygen might be conveyed to the catalyst through porous substrates on which a wet layer of photocatalyst is deposited (**Figure** **7**). Microporous geometries are especially beneficial for increasing the contact area between the gas and the solid‐liquid phase.

However, there are only a few examples of micro‐engineered gas diffusion layers for ORR. In one of the reported attempts, a melamine foam was chosen as a porous substrate and as a support for photocatalytic graphitic carbon nitrides. Faster gas diffusion to the reaction center led to a 10‐fold increase in H_2_O_2_ production over the traditional biphasic system.^[^ ^97^ ^]^ In general, for triphasic reactors, it is fundamental to design hydrophobic catalysts. Hydrophobicity will not only favor H_2_O_2_ desorption but will also ensure the high stability of the catalyst at the liquid/air interface. Materials whose chemical composition can be tuned through bottom‐up approaches like covalent organic frameworks (COFs) are good candidates for this purpose.^[^ ^98^ ^]^ Reticulate materials including metal‐organic frameworks are also characterized by high surface area for improved reactant/product diffusion.

Finally, ORR admittedly still strongly relies on organic alcohols or amines as sacrificial electron donors. The whole field of photoredox catalysis needs a paradigm shift from the convenient but wasteful optimization of half‐reactions to the co‐valorization of both oxidation and reduction sides. The latter perspective has risen in recent years under the name of “photoreforming”. Although this is majorly referred to as biomass valorization,^[^ ^99^ ^]^ a whole range of non‐sacrificial electron donor substrates exists. These include ethylene glycol from plastic bottle recycling,^[^ ^100^ ^]^ glycerol from the synthesis of biodiesel,^[^ ^86^ ^]^ 5‐hydroxymethylfurfural (HMF) from the degradation of various biomass‐derived substrates.^[^ ^101^ ^]^

### 3.2 Proposed Mechanisms for Light‐Driven H_2_O_2_ Synthesis

Clarifying the chemical pathways of hydrogen‐oxygen combination in either ORR or WOR is fundamental for the design of photo and electrocatalysts with enhanced selectivity for hydrogen peroxide. The mechanism of the anthraquinone process is well‐understood and it is known to proceed via a radical autoxidation in which intermediate HOO· species are generated as a result of hydrogen atom transfer (HAT) from hydroquinone by dioxygen. Two subsequent HATs lead to H_2_O_2_ and the oxidized anthraquinone.^[^ ^77^ ^]^

**Figure 8:**
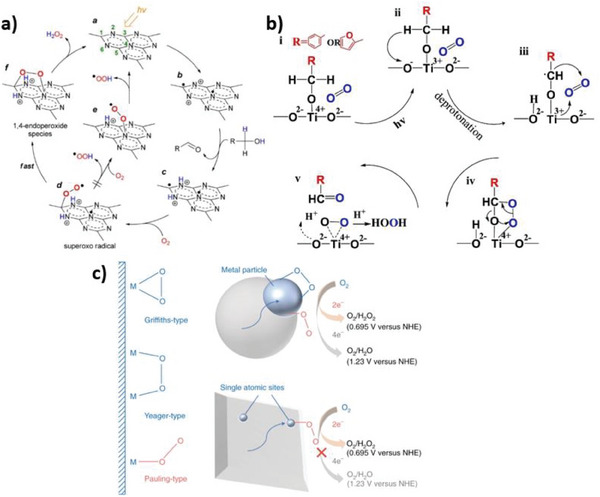
a) Scheme of the proposed mechanism of selective 2e^−^ ORR on C_3_N_4_. Reproduced with permission.^[^ ^81^ ^]^ Copyright 2014, American Chemical Society; b) Scheme of the proposed mechanism of selective 2e^−^ ORR on TiO_2_ using aromatic alcohols as electron donors. Reproduced with permission.^[^ ^102^ ^]^ Copyright 2020, Elsevier; c) Illustration of the edge‐on and end‐on absorption of O_2_ on catalysts and suppressed 4e^−^ reduction on single atoms. Reproduced with permission.^[^ ^103^ ^]^ Copyright 2021, Springer Nature.

Carbon nitrides are excellent catalytic substrates for 2e^−^ ORR thanks to the formation of a favorable 1,4‐endoperoxo species on triazine units. This labile intermediate first detected with Raman spectroscopy by Shiraishi et al. is expected to be key to suppressing 1e^−^ ORR to hydroperoxyl radicals.^[^ ^81^ ^]^ Through the formation of the peroxo bridge, two oxygen atoms can receive two electrons and two protons simultaneously and directly from the C_3_N_4_ scaffold (**Figure** **8**a). The two electrons are supposed to be localized at the C1 and N4 positions of the triazine ring, while holes are at the N2 and N6 positions. Once the holes oxidize an electron donor (such as CH_3_OH), oxygen is reduced generating a peroxo species. While this intermediate would generally be protonated and released as HOO·, the proximity of a highly reducing electron on the triazine kinetically favors the formation of the 1,4‐endoperoxide, and thus the subsequent protonation and desorption as H_2_O_2_.

A similar involvement of peroxo species was proposed for TiO_2_‐photocatalyzed H_2_O_2_ production (Figure 8b).^[^ ^102^ ^]^ Here, electron‐donating deprotonated alcohols form a complex with surface Ti^IV^ centers. Upon UV irradiation, titanium is reduced to Ti^III^ and then a hydrogen atom is transferred from the alcohol to a surface oxygen atom, yielding an alkyl radical. Dioxygen swiftly binds to this reactive species and to Ti, resulting in a five‐membered ring intermediate. Finally, bonds rearrange resulting in an aldehyde and a cyclic Ti‐O‐O peroxide, which is selectively converted to H_2_O_2_ by double protonation. Such a mechanism was proposed for aromatic alcohols, which better stabilize the alkyl radical intermediate. The chemical adsorption of alcohols is also beneficial in inhibiting UV‐catalysed hydrogen peroxide decomposition on TiO_2_.

Recently, mechanistic advances reported by Ohno and co‐workers suggested that single‐atom catalysts (SACs) might have a great potential to suppress 4‐e^−^ ORR to water.^[^ ^103^ ^]^ Conversion of O_2_ to H_2_O inevitably proceeds through the cleavage of the O‐O bond. Oxygen can adsorb on ORR catalysts in different geometries that could favor or prevent bond scission, namely edge‐on – including Griffiths‐type and Yeager‐type – and end‐on, or Pauling‐type (Figure 8c). The former – that incidentally pertains to the endoperoxide species described above – induces strain in the adsorbate structure so that the O‐O bond is more likely to break. This configuration is common on metals or alloys, where all adjacent atoms are possible adsorption centers. Conversely, in SACs, catalytic atoms are isolated, so that dioxygen can only attach in an end‐on fashion. Therefore, the scission of the two oxygen atoms is less favored, and conversion to water is suppressed. This was demonstrated on single‐atom Sb supported on C_3_N_4_.^[^ ^103^ ^]^

**Figure 9:**
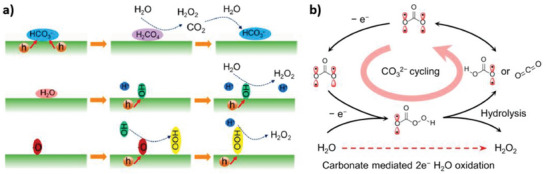
a) Schemes of proposed mechanisms for 2e^−^ WOR. Reproduced with permission.^[^ ^88^ ^]^ Copyright 2019, American Chemical Society; b) Scheme of proposed mechanism for HCO_3_^−^‐assisted H_2_O_2_ electrochemical synthesis. Reproduced with permission.^[^ ^104^ ^]^ Copyright 2022, Springer Nature.

As far as the WOR approach is concerned, more investigations are needed to unveil the most likely chemical pathways (**Figure** **9**a). Catalyst design should focus on the fate of intermediate HOO· species generated from OH^−^. If deprotonation is favored, then HOO· ends into the thermodynamically more stable O_2_, but if protonation is instead enhanced – for instance kinetically from adsorbed H^+^ – H_2_O_2_ is the preferred product. Nevertheless, efforts dedicated to 2e^−^ WOR should definitely start from the critical and unique HCO_3_^−^‐based catalysis. As illustrated in Figure 9b, percarbonate (HCO_4_^−^) ions were detected as key intermediates for the electrochemical oxidation of water to hydrogen peroxide.^[^ ^104^ ^]^ It was proposed that bicarbonate ions first undergo step‐wise 2e^−^ oxidation and addition to OH^−^. It is therefore important to select anodes to enable efficient electron extraction from carbonate ions. In the future, applications for catalytic green H_2_O_2_ synthesis from the water will probably be in the form of electrocatalytic or photo electrocatalytic cells, with HCO_3_^−^ anolytes constituting a fundamental component.

Overall, it can be concluded that major advances in sustainable hydrogen peroxide production will come from systems capable of combining ORR and WOR in a single clean and non‐sacrificial dual‐cycle. Few attempts have been successfully proven, so far. A possible approach consists of including isolated oxidative and reductive centers on a common substrate. This strategy was demonstrated by Kim et al. in the combination of reducing Co single atoms and oxidizing grafted anthraquinone molecules on a C_3_N_4_ support.^[^ ^105^ ^]^ Despite the ideal photocatalytic process coupling ORR and WOR still far from the efficiency displayed by the commercial fossil‐based H_2_O_2_ anthraquinone process, the H_2_O_2_ photocatalytic production deserves the spotlight in terms of solar to chemical approach. The photocatalytic production of H_2_O_2_ in some optimized conditions, for example, employing sacrificial reagents (e.g., biomass‐derived molecules or wastes) can display impressively high H_2_O_2_ production rates.^[^ ^95^ ^]^ State‐of‐the‐art TiO_2_ achieved a remarkable H_2_O_2_ production rate of 3.3 mM h^−1^, although requiring illumination at shorter wavelengths (> 280 nm) and benzyl alcohol as an electron donor,^[^ ^106^ ^]^ but a very similar performance was demonstrated with C_3_N_4_ composites including pyromellitic diimide (PDI) and reduced graphene oxide (rGO) in the presence of widely available ethanol.^[^ ^107^ ^]^ Both these materials also achieved remarkable apparent quantum yields (AQY) of 29% and 7%, respectively. In this sense, this process might be one of the examples of direct use of photocatalysis in a solar‐to‐chemical approach, closest to reaching the efficiency required to be commercially used. Certainly, more advances are still needed, as well as investigations about the economic feasibility. However, it is worth highlighting the potential of this technology, which might be disruptive to the production of an important chemical responsible for an important consumption of fossil‐derived H_2_.

## 4 Photocatalytic‐driven Hydrogenation

Another crucial aspect of the industrial chemical transformation is the reduction of organic compounds. This particular field stands out as one of the most prevalent in industrial reactions, with an estimated 25% of all industrial processes incorporating at least one hydrogenation step.^[^ ^108^, ^109^ ^]^ In addition to its extensive application in fine chemicals and pharmaceuticals, hydrogenation is also widely employed in petrochemicals, particularly during hydrodenitrogenation and hydrodesulfurization steps.^[^ ^110^ ^]^

Traditionally, the industrial sector has made use of noble metals as catalysts and molecular hydrogen as the primary agent for reducing unsaturated bonds, such as C═ C, C═N, and C═O, this approach is not only expensive due to the rarity of the noble metals, but also hazardous for the high pressures and temperatures of operation. In recent times, a new approach to hydrogenation has gathered attention, known as transfer hydrogenation. This strategy involves an alternative hydrogen donor for the reduction process without the employment of H_2_ gas. Transfer hydrogenation offers unique advantages by providing opportunities for a more sustainable process, readily available, inexpensive donors, and its versatility, as it avoids the challenge associated with H_2_ storage and production. Typically, this reduction is conducted catalytically and driven by thermal or light energy.

**Figure 10:**
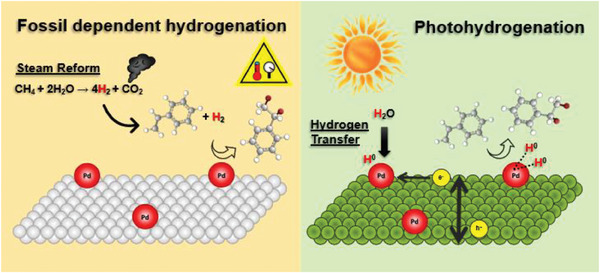
Comparative illustration between the industrial hydrogenation process, which relies on molecular hydrogen derived from methane reforming, and the photocatalytic process utilizing hydrogen transfer from water as an alternative source.

Nevertheless, several studies have demonstrated the high potential of photocatalytic systems in comparison to the thermal pathway. Beyond its compelling sustainability proposition, photohydrogenation offers highly efficient and inherently more energetically favorable reaction mechanisms that outperform traditional thermal pathways,^[^ ^111^ ^]^ reducing the reliance on fossil‐derived hydrogen (**Figure** **10**). One of the key advantages of photocatalytic transfer hydrogenation is the ability to harness solar energy for the reduction process, making it a sustainable and environmentally friendly approach. Additionally, the controlled application of moderate heat in combination with photohydrogenation can lead to synergistic effects, further improving reaction efficiency and selectivity.^[^ ^112^ ^]^

This provides the engineering of new catalysts with rationalization capabilities to design and produce highly efficient systems, wherein hot photogenerated charges are the reduction sites within the process. This session will delve into the multitude of advantages and promising prospects offered by tailored photocatalytic processes, specifically aimed at transfer hydrogenation reactions. In other words, the photocatalytic hydrogen transfer reaction is a perfect example of the direct use of green hydrogen through a solar‐to‐chemical strategy. We have also compiled a set of the most influential works involving hydrogen transfer reactions using photocatalysts (**Table** **3**).

**Table 3:** Summary of different photocatalysts applied for transfer hydrogenation of unsaturated bonds.

Catalyst	Hole scavenger employed	Catalyst mass [mg]	Light wavelength [nm]	Target molecule/ product	Reaction time [h]	Conversion/ Selectivity [%]	Reference
Ni/C_3_N_4_	TEA	3	420	Phenylacetylene/Styrene	14	100 (97)	[114]
Pt/Nb_2_O_5_‐A	EtOH	10	365	Phenylacetylene/Styrene	10	≈60 (N/A)	[115]
Fe/g‐C_3_N_4_	i‐PrOh (10%)	20	365	Nitrobenzene/Aniline	3	100 (100)	[116]
Pd/Si	HCOOH	25	375–800	Nitrobenzene/Aniline	3	100 (99)	[118]
Au@ZrO_2_	HCOOH	50	400–750	Phenylacetylene/Styrene	16	100 (25)	[119]
Pt/g‐C_3_N_4_	TEA (10%)	30	420	HMF/DHMF	4	0.457 h^−1^ (TOF)^a)^	[122]
Pd‐mpg‐C_3_N_4_	H_2_O	10	427	Styrene/Ethylbenzene	10	100 (100)	[124]
Ni‐N/CN	H_2_O	20	420	2‐Ethynylnaphthalene/ 2‐vinylnaphthalene	2	100 (98)	[125]
Pd/TiO_2_	Glucose	25	350	4‐nitroacetophenone/ 4‐aminoacetophenone	12	99 (99)	[126]
TiO_2_/Ce_2_S_3_	H_2_O	15	365	Nitrobenzene/Aniline	1.5	100 (99)	[127]
LCN	H_2_O	20	420	4‐nitrophenol/4‐aminophenol	1.67	96.5 (98.9)	[128]

^a)^ Turnover frequency (TOF).

Moving away from H_2_ as a chemical feedstock for organic synthesis is not a trivial task, as performing hydrogenations with H_2_ offers a perfect atom efficiency reaction. Regardless, the current need for reactions in mild conditions and with green hydrogen sources has pushed research toward alternative hydrogen sources. Several types of sacrificial donors and catalytic systems have been studied as hydrogen sources for transfer hydrogenation reactions.^[^ ^109^, ^113^ ^]^ In these cases, for the chemical species to be considered as relevant substitutes for H_2_ in large‐scale applications, they must be cheap, easy to transport, stable, and renewable. Additionally, they must be paired with highly active catalysts to have competitive processes that could pose as a viable route for hydrogenation.

One promising class of compounds that can be employed as hydrogen sources in H‐transfer reactions are organic molecules derived from biomass (e.g., alcohols, and carboxylic acids). They possess low toxicity and are widely available and cheap. Small‐chain alcohols such as methanol and ethanol have been previously used in semihydrogenation of alkynes, with great control in selectivity. For instance, Jia et al. supported nickel nanoparticles on a carbon nitride scaffold, which was successful in the photocatalytic semihydrogenation of phenylacetylene into styrene, with selectivity >90% over several usages.^[^ ^114^ ^]^ Meanwhile, Su and coworkers demonstrated that by inducting modifications in structure of an Nb_2_O_5_ support and the Pt species on it, they could control the products in the photoreduction of phenylacetylene. On Pt/2D Nb_2_O_5_ hydrogen transfer from ethanol led to styrene as the major product, while Pt/Bulk Nb_2_O_5_ was more selective to H_2_ evolution.^[^ ^115^ ^]^ Isopropanol has also been used as a hydrogen source in the reduction of nitroarenes in the work of Deng et al. The authors used non‐noble Fe‐based photocatalysts (Fe/C_3_N_4_) in which iPrOH was oxidized into acetone and nitrobenzene reduced into acetone with no reported H_2_ evolution.^[^ ^116^ ^]^

Formic acid (FA) is regarded as one of the best hydrogen carries in terms of gravimetric capacity, safety, and cost.^[^ ^117^ ^]^ Tsutsumi et al. have demonstrated a photocatalytic system utilizing a Pd/Si hybrid material for the reduction of several nitroarenes in ambient conditions with great yields.^[^ ^118^ ^]^ Another great example was demonstrated by Huang et al. where FA was successfully employed in the reduction of nitroarenes, olefins, aldehydes, imides, and alkynes, by the photocatalytic activity of Au@ZrO_2_ nanocatalyst.^[^ ^119^ ^]^

**Figure 11:**
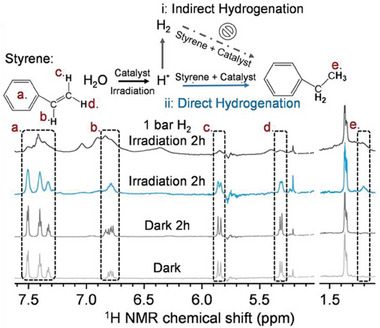
Operando ^1^H‐NMR of the photocatalytic water transfer hydrogenation of styrene induced by the photocatalyst Pd_1_‐mpg‐C_3_N_4_. Reproduced with permission.^[^ ^124^ ^]^ Copyright 2022, John Wiley and Sons.

Water would be regarded as the most ideal hydrogen source for transfer reactions; however, the reaction pathways are usually impeded by the energy‐demanding and kinetic hindered water oxidation reaction. Luckily, the attractiveness of this approach has led to intensive research in the past years, and significant progress has been made in this field by both homogeneous and heterogeneous photocatalysts.^[^ ^120^, ^121^ ^]^ In 2016 Guo et al. managed to demonstrate a photocatalytic system where Pt/g‐C_3_N_4_ catalyst was able to reduce water into hydrogen and subsequently utilize the H_2_ for the reduction of HMF into DHMF.^[^ ^122^ ^]^ There are many cases where hydrogen is directly consumed in the reaction, in the form of H•.^[^ ^123^, ^124^ ^]^ This sophisticated approach was thoroughly investigated by Zhao and collaborators, in their work a Pd_1_‐mpg‐C_3_N_4_ was used for the photo‐induced hydrogenation of styrene by water. They monitored the progress of the reaction employing operando ^1^H NMR and proved that no H_2_ was being produced, instead protons where directly added to the molecule (**Figure** **11**).^[^ ^124^ ^]^ The selective semihydrogenation of alkynes by water‐sourced proton was also explored in the work of Arcudi et al. by combining a heterogeneous photosensitizer of mpg‐C_3_N_4_ and Co‐TPPS as the catalytic site. The authors managed to convert acetylene into ethylene with 99% selectivity even in competitive conditions (ethylene co‐feed).^[^ ^121^ ^]^ Meanwhile, Jie et al. managed to carry the deuteration of several alkynes into their respective alkenes using D_2_O as a D‐source using Ni‐N/C_3_N_4_ photocatalyst under ambient conditions and with great selectivity.^[^ ^125^ ^]^

**Figure 12:**
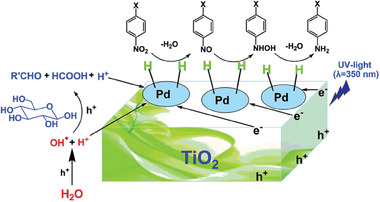
Proposed reaction pathway in the selective photocatalytic transfer hydrogenation of nitroarenes into anilines by an H_2_O/glucose system catalyzed by Pd/TiO_2_. Reproduced with permission.^[^ ^126^ ^]^ Copyright 2016, Royal Society of Chemistry.

The synthesis of anilines is of great industrial significance, a green and renewable route is highly desirable. Taking that into account, Zhao and coworkers developed a Pd/TiO_2_ photocatalyst capable of a transfer hydrogen reaction. In their work, a water/glucose solution was used as an H‐source for the reduction of several nitroarenes under UV‐light. The results showed that both glucose and water acted as H‐sources (**Figure** **12**), with water being responsible for ≈24% of hydrogen delivery.^[^ ^126^ ^]^ In another work, Xu et al. utilized TiO_2_ support with Cd_2_S_3_ nanoparticles for direct nitrobenzene reduction by water, in their most optimized condition, their hybrid photocatalyst was capable of producing aniline at 99% selectivity under 90 minutes.^[^ ^127^ ^]^ A carbon nitride‐based catalyst was also investigated in this type of reaction by Yao et al. In their work, p‐nitrophenol was reduced into p‐aminophenol by Pt/C_3_N_4_ and water, while simultaneously H_2_ was also being produced by the water splitting reaction. The authors combined the use of DFT calculations and isotope labeling to prove that H° coming from water was the reactive species for the reduction.^[^ ^128^ ^]^

Deuterium labeling in organic molecules is another chemical transformation of great interest due to the kinetic isotope effect, allowing investigation of reaction mechanisms,^[^ ^129^ ^]^ modification of selectivity,^[^ ^130^ ^]^ and change in physical‐chemical properties that can be exploited for pharmaceutical compounds.^[^ ^131^ ^]^ In that sense, transfer deuteration from water offers a direct and waste‐free synthetic approach for high‐end fine chemicals. Aryl halides are the most common substrates for this kind of transformation,^[^ ^132^, ^133^, ^134^ ^]^ for instance, Liu et al. used a porous CdSe photocatalyst for the deuteration of different classes of aryl halides. The photocatalytic system was even capable of deuteration of C─Cl and C─F bonds with selectivity yields >90%.^[^ ^133^ ^]^ Another great example of deuteration of the highly stable C‐Cl bonds has been demonstrated by Ling et al., where Pd nanosheets supported on crystalline polymeric carbon nitrides (CPCN) were able to induce the deuterium transfer form D_2_O using triethanolamine (TEOA) as a sacrificial reagent, without leading to reduction or hydrogenation of other functional groups.^[^ ^132^ ^]^ Besides aryl halides, diaryl alcohols, and carbonyl are also viable choices of substrate for transfer deuteration,^[^ ^135^ ^]^ Nan and coworkers managed to utilize diaryl alcohols as substrate for deuterated water transfer reactions. Their approach involved the usage of CdSe quantum dots under ambient conditions and visible light irradiation. This reaction system was successful in the deuteration of a great scope of substrate with good yields.^[^ ^136^ ^]^

## 5 Photocatalytic‐driven CO_2_ Reduction

Our strong reliance on fossil fuels gives rise to innumerable challenges. Not only are they finite resources, but their combustion exerts a deep impact on the sustainability and thermal equilibrium of our planet.^[^ ^137^ ^]^ Carbon dioxide stands out as a primary driver of these anthropogenic transformations, attracting great efforts toward the development of new strategies and technologies aimed at mitigating its occurrence and reducing atmospheric concentrations. Despite the environmental concerns surrounding it, CO_2_ is widely recognized as an optimal feedstock for the conversion into C_1_ and C_2+_ high‐demand chemicals.^[^ ^138^, ^139^, ^140^ ^]^ Various catalytic processes, including CO_2_ hydrogenation, Fischer‐Tropsch, water‐gas‐shift, and reverse‐water‐gas‐shift represent the primary routes for carbon valorization. Conventionally, these processes are performed utilizing fossil‐derived H_2_ as a reductive agent and expensive supported metal catalysts operating under harsh conditions.^[^ ^140^ ^]^ CO_2_ hydrogenations employing fossil‐derived H_2_ are not neutral in CO_2_ emissions, once H_2_ production by steam reforming or gasification holds an important CO_2_ footprint. Hence, it is essential to develop new strategies to convert carbon dioxide to valuable chemicals employing green hydrogen sources).

**Figure 13:**
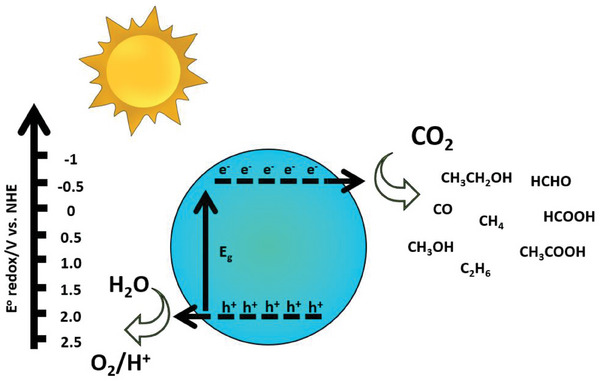
Diagram representing the photoreduction of CO_2_ using water as electron donor.

More recently, in pursuit of a more environmentally friendly and feasible method, the scientific community has been making substantial efforts to mimic the natural photosynthetic process by exploring photocatalytic approaches induced by solar energy (**Figure** **13**). Essentially, the photocatalytic CO_2_ conversion presents fundamental limitations primarily associated with the high stability of the CO_2_ molecule, low product selectivity, and slow kinetics involved in the multi‐step reduction toward more complex products.^[^ ^138^, ^141^ ^]^ Moreover, the band structure of typical semiconductors is not adequate for simultaneous CO_2_ reduction and H_2_O oxidation, further challenging the process (See below the potential table measured at pH 7).^[^ ^142^ ^]^ Nevertheless, remarkable progress has been made in this field, being close to the 10% efficiency required for artificial photosynthesis to be feasible.^[^ ^143^ ^]^

(13) $${\mathrm{CO}}_{2(g)}+e^{-}\to {\mathrm{CO}}_{2}^{•-}\left(-1.90\;V\;\mathrm{vs}\;\mathrm{NHE}\right)$$

(14) $${\mathrm{CO}}_{2(g)}+2H_{\left(\mathrm{aq}\right)}^{+}+2e^{-}\to {\mathrm{CO}}_{\left(g\right)}+H_{2}O_{\left(l\right)}\left(-0.53\;V\;\mathrm{vs}\;\mathrm{NHE}\right)$$

(15) $${\mathrm{CO}}_{2(g)}+2H_{\left(\mathrm{aq}\right)}^{+}{+2e}^{-}\to {\mathrm{HCOOH}}_{\left(\mathrm{aq}\right)}\;\left(-0.61\;V\;\mathrm{vs}\;\mathrm{NHE}\right)$$

(16) $$\begin{array}{c} {\mathrm{CO}}_{2(g)}+4H_{\left(\mathrm{aq}\right)}^{+}+4e^{-}\to {\mathrm{HCHO}}_{\left(\mathrm{aq}\right)}+H_{2}O_{\left(l\right)}\left(-0.48\;V\;\mathrm{vs}\;\mathrm{NHE}\right) \end{array}$$

(17) $$\begin{array}{c} {\mathrm{CO}}_{2(g)}+6H_{\left(\mathrm{aq}\right)}^{+}+6e^{-}\to {\mathrm{CH}}_{3}{\mathrm{OH}}_{\left(\mathrm{aq}\right)}+H_{2}O_{\left(l\right)}\;\left(-0.38\;V\;\mathrm{vs}\;\mathrm{NHE}\right) \end{array}$$

(18) $$\begin{array}{c} {\mathrm{CO}}_{2(g)}+8H_{\left(\mathrm{aq}\right)}^{+}+8e^{-}\to {\mathrm{CH}}_{4(\mathrm{aq})}+2H_{2}O_{\left(l\right)}\;\left(-0.24\;V\;\mathrm{vs}\;\mathrm{NHE}\right) \end{array}$$

(19) $$\begin{array}{c} 2CO_{2(g)}+8H_{\left(\mathrm{aq}\right)}^{+}+8e^{-}\to {\mathrm{CH}}_{3}{\mathrm{COOH}}_{\left(\mathrm{aq}\right)}+2H_{2}O_{\left(l\right)}\;\left(-0.31\;V\;\mathrm{vs}\;\mathrm{NHE}\right) \end{array}$$

(20) $$\begin{array}{c} 2CO_{2(g)}+14H_{\left(\mathrm{aq}\right)}^{+}+14e^{-}\to C_{2}H_{6(\mathrm{aq})}+4H_{2}O_{\left(l\right)}\;\left(-0.51\;V\;\mathrm{vs}\;\mathrm{NHE}\right) \end{array}$$

The photoreduction of CO_2_ remains a challenging reaction due to its inherent complexity. In essence, there are three primary obstacles that limit the progress of this reaction. Firstly, the adsorption and activation of CO_2_ pose a significant challenge. Secondly, there are economic concerns associated with the use of expensive sacrificial reagents. Lastly, the formation of higher‐value products, such as oxygenates, presents additional difficulties.

### 5.1 CO_2_ Activation

Regarding the carbon dioxide activation, in photocatalytic systems, it is often observed that CO_2_ molecules react with photogenerated electrons, leading to the formation of CO_2_^•−^ species (Equation 13) that can undergo further conversions. This process is recognized as the rate‐limiting step in photocatalytic CO_2_ reduction reactions due to its associated high negative potential (−1.9 V vs NHE). To overcome this challenge, one strategy involves the adsorption of CO_2_ molecules onto the surface of the photocatalyst, resulting in the formation of partially charged CO_2_^δ•-−^ species.^[^ ^144^ ^]^ This process effectively lowers the energy barrier for electron transfer, facilitating subsequent reactions.

**Figure 14:**
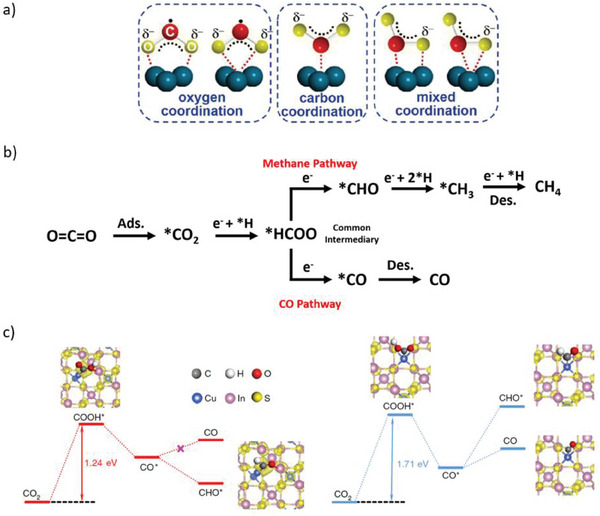
a) Schemes illustrating the three possibilities of CO_2_ activation on the catalyst surface. Reproduced with permission.^[^ ^146^ ^]^ Copyright 2020, Royal Society of Chemistry; b) Scheme representing the different pathways proposed for CO_2_ reduction; c) Free energy of CO_2_ reduction using dual‐site Cu‐In to manipulate the CO and CH_4_ production induced by controlled sulfur structural vacancies. Reproduced with permission.^[^ ^147^ ^]^ Copyright 2019, Springer Nature.

Various reactive pathways to absorb CO_2_ molecules have already been firmly established in the literature.^[^ ^145^ ^]^ Due to the remarkable stability associated with the linear arrangement of the CO_2_ molecule, the pivotal step involves breaking this linearity by activating the molecule during its interaction with the catalyst surface. The direct reduction of the gaseous phase CO_2_ requires high reductive potential, making this process unfeasible (Equation 13). However, once chemisorbed in the active site of the photocatalyst, low energy potentials arise, turning the reaction kinetically more viable. Consequently, the catalyst design becomes an immensely crucial aspect to consider in CO_2_ reduction, as it serves as a modulator for achieving a specific product. The diverse proposed mechanisms exhibit distinct characteristics depending on the geometry in which the CO_2_ molecule is activated on the catalyst surface. Hence, following a specifical adsorption orientation, a series of successive electron transfer processes ensues (**Figure** **14**b). Chemisorption can occur through three intriguing configurations (Figure 14a): i) pair acceptors, typically involving metals that interact with the lone pairs of terminal oxygen; ii) similarly, sites rich in electron density are capable of donating electrons to the vacant carbon orbitals, coordinating with them; iii) lastly, simultaneous coordination occurs through both carbon and oxygen atoms.

Several strategies have been developed to enhance CO_2_ adsorption, including defect engineering involving oxygen, anion, and cation vacancies, surface modification, doping, and stabilization of single atoms (**Table** **4**). Luo and Ji found that oxygen vacancies on TiO_2_ exhibit higher activity in CO_2_ reduction compared to surface Ti atoms.^[^ ^148^ ^]^ This enhanced activity can be attributed to the combination of fast hydrogenation and deoxygenation pathways promoted by these lattice defects. The presence of oxygen vacancies in the lattice facilitates the deoxygenation of CO_2_, leading to the formation of CO* or hydrogenation toward COOH* species. Dong and colleagues demonstrated that the introduction of Cu_2_O onto ceria results in the creation of additional oxygen defects and Ce^3+^ sites, which play a crucial role in the deoxygenation of CO_2_* to generate CO molecules.^[^ ^149^ ^]^ The hydrogenation of CO_2_* molecules to COOH* species also contributes to the production of CO. Xie et al., for instance, developed BiOBr atomic layers with controlled oxygen vacancies and observed that the charge delocalization at these sites contributes to the formation of COOH* intermediates, which enhances the CO generation 20 and 24‐fold than non‐defect and bulk BiOBr, respectively.^[^ ^150^ ^]^ Also, the introduction of desired defects could control the selectivity to other compounds, for example, sulfur vacancies on CuIn_5_S_8_ layers change the reaction pathway promoting the hydrogenation of CO* intermediates to produce CHO*, leading to high selectivity to CH_4_ instead of CO molecules (Figure 14c).^[^ ^147^ ^]^ Recently, Long and collaborators showed that the construction of twining defects on Au NPs supported on TiO_2_ could improve the selectivity toward CO than regular Au‐TiO_2_ and bare TiO_2_ materials.^[^ ^151^ ^]^

**Table 4:** Summary of different photocatalysts applied for CO_2_ reduction.

Catalyst	Hole scavenger employed	Catalyst mass [mg]	Light wavelength [nm]	Main product	Rate conversion [µmol g^−1^ h^−1^]	AQY [%]	Reference
2% CuCe	H_2_O	50	Xenon lamp	CO	0138	N/A	[149]
BiOBr	H_2_O	100	>400	CO	84.4	N/A	.^[^ ^150^ ^]^
V_S_‐CuIn_5_S_8_	H_2_O	N/A	420‐	CH_4_	8.7	0.786^c)^	.^[^ ^147^ ^]^
5‐T‐Au/TiO_2_	H_2_O	10	Xenon lamp	CO	608	0.2 (380 nm)	[151]
Bi‐OV‐α‐Ga_2_O_3_	H_2_O	10	Xenon lamp	CO	42.3	N/A	[152]
RuP/C_3_N_4_	TEOA (20%)	8	>400	HCOOH	141 (TON)^a)^	5.7 (400 nm) 0.9 (420 nm)	[153]
RuRu′/Ag/C_3_N_4_	TEOA (20%)	4	Hg lamp	HCOOH	3110 (TON)^b)^	5.2 (400 nm)	[154]
Fe SAS/Tr‐COF	TEOA	5	>420	CO	980.3	3.17 (420 nm)	[155]
Cu‐CCN	CH_3_CH_2_OH	25	Xenon lamp	CO	3.086	N/A	[156]
OF‐Co	TEOA (20%)	2	400‐	CO	200.6	N/A	[157]
Ni‐TpBpy	TEOA (20%)	10	>420	CO	915	0.3 (420 nm)	[158]
Ni‐SA/ZrO_2_	H_2_O	10	Xenon lamp	CO	11.8	0.92% (365 nm) 0.36% (420 nm)	[159]
CoRu‐HCNp	CH_3_CH_2_OH	25	Xenon lamp	CO	27.31	2.8% (385 nm)	[160]
α‐Fe2O3/g‐C_3_N_4_	H_2_O	25	Xenon lamp	CO	27.2	0.49% (365 nm) 0.96% (420 nm)	[161]
0.33AB	H_2_O	10	Xenon lamp	CO	212.6	N/A	[163]
P@U	H_2_O	5	>420	HCOOH	146.0	N/A	.^[^ ^164^ ^]^
TTCOF‐Zn	H_2_O	100	420‐	CO	2.05	N/A	[166]
TCOF‐MnMo_6_	H_2_O	2	400‐	CO	33.19	0.0067% (400 nm)	[167]
BiOBr_0.6_Cl_0.4_	H_2_O	10	Xenon lamp	CO	15.86	N/A	[168]
Cu SAs/UiO6‐NH_2_	TEOA	100	>400	MeOH/EtOH	5.33/4.22	N/A	[169]
PTh/Bi_2_WO_6_	H_2_O	50	>420	MeOH/EtOH	14.1/5.1	0.0086% (475 nm)	[170]
Co/g‐C_3_N_4_.2 SAC	H_2_O	5	Xenon lamp	MeOH	235.5	N/A	[171]
P/Cu SAs@CN	TEOA (10%)	≈0.5	Xenon lamp	MeOH	616.6	12.55% (350 nm)	[172]
Ti_0.91_O_2_‐SL	H_2_O	10	Xenon lamp	CO	67.0	0.48% (385 nm) 0.15% (415 nm) 0.06% (520 nm)	[173]
VS‐SAL_10_	H_2_O	5	Xenon lamp	C_2_H_4_	44.3	0.51% (415 nm)^b)^	[174]

^a)^ Values of turnover number (TON);

^b)^ External quantum efficiency (EQE);

^c)^ Full spectrum.

Another important approach to improve CO_2_ activation is surface modification with organic molecules, functional groups, or cocatalysts. For example, the introduction of bismuth nanoparticles (Bi NPs) on α‐Ga_2_O_3_ significantly improves the selectivity of CO_2_ to CO (10% for bare α‐Ga_2_O_3_ vs 80% for Bi‐α‐Ga_2_O_3_) by avoiding H_2_ evolution, due to the intrinsic weak adsorption of H* species of Bi NPs, and increasing the energy barrier of the CH_2_* intermediate, related to CH_4_ evolution.^[^ ^152^ ^]^ Moreover, ruthenium complexes immobilized on carbon nitride structures boost the photoreduction of CO_2_ to formic acid with an impressive turnover number (>1000) and apparent quant yield (5.7%) using visible light (400 nm) and TEOA as sacrificial agent.^[^ ^153^ ^]^ Later, Maeda et al. revealed that binuclear Ru(II) complexes supported on a system consisting of Ag/C_3_N_4_ significantly enhance HCOOH production with an even higher turnover number (>33 000) and elevate selectivity.^[^ ^154^ ^]^

**Figure 15:**
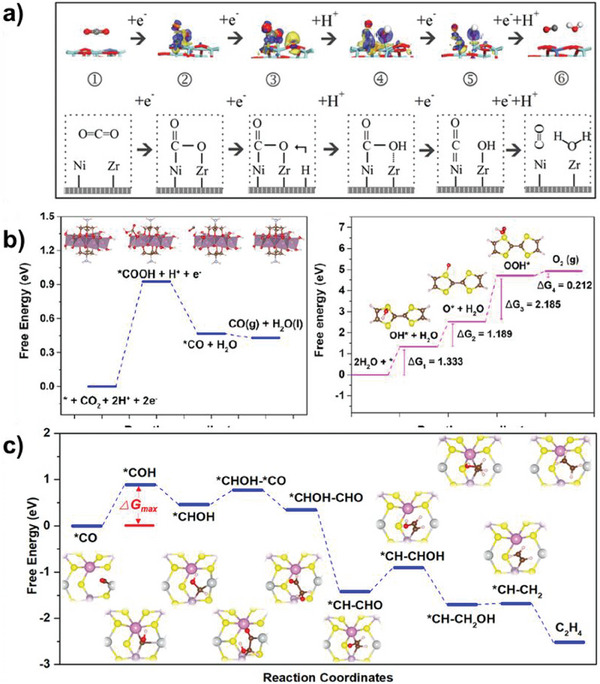
a) Differential charge density diagrams and intermediates during CO_2_ reduction to CO over Ni‐SA/O model. Reproduced with permission.^[^ ^159^ ^]^ Copyright 2020, John Wiley and Sons; b) Free energy diagrams for CO_2_ reduction to CO on MnMo_6_ and for H_2_O oxidation to O_2_ on TTF of TCOF‐MnMo_6_. Reproduced with permission.^[^ ^167^ ^]^ Copyright 2022, American Chemical Society; c) Gibbs free energy diagrams for CO reduction to C_2_H_4_ over AgInP_2_S_6_ with sulfur vacancies. Reproduced with permission.^[^ ^174^ ^]^ Copyright 2021, Springer Nature.

Another notable approach for enhancing CO_2_ activation and consequent reduction involves the stabilization of single atoms (SAs),^[^ ^155^ ^]^ which can significantly enhance metal utilization and selectivity to a desirable product. Copper SAs supported on crystalline carbon nitrides have proven to be effective in achieving nearly 100% selectivity in carbon monoxide production.^[^ ^156^ ^]^ The authors have elucidated that selectivity control is achieved by lowering the energy barrier of the deoxygenation pathway through the presence of isolated copper sites, resulting in the predominant formation of CO as the product. Moreover, the combination of single atoms (SAs) with photocatalytic systems improves CO_2_ activation. For instance, Ye et al. reported that Co single atoms supported on metal‐organic frameworks not only enhance charge separation through electron migration to the metal center but also reduce the energy barrier for the deoxygenation of CO_2_* to CO*.^[^ ^157^ ^]^ In addition, SAs can synergistically interact with the support material to enhance CO_2_ photoreduction. The synergistic effect of Ni single atoms and the covalent organic framework (COF) structure to enhance CO production was demonstrated by Zou et al.^[^ ^158^ ^]^ The authors suggested that the keto units present in the COF structure contribute to stabilizing COOH* intermediates, which can undergo further dehydration to generate CO. Additionally, single atoms can serve as activation centers in conjunction with atoms on the support material, leveraging their distinct catalytic abilities to function in unique ways. For instance, Ni SAs supported on defect‐rich ZrO_2_ promote the stabilization of COOH* species (**Figure** **15**a), which can be protonated to produce CO molecules.^[^ ^159^ ^]^ The combination of different metal activation centers has garnered interest within the research community. Furthermore, dual‐single‐atom materials have emerged as intriguing catalysts, as the metallic centers collaborate to enhance photocatalytic CO_2_ reduction. Xiang et al. demonstrated that Co and Ru dual‐single‐atoms can synergistically improve charge transfer and facilitate CO_2_ reduction to both CO and CH_4_.^[^ ^160^ ^]^ This dual‐metallic system is able to separate charges with the assistance of Co sites while Ru centers promote CO_2_ chemisorption.

### 5.2 CO_2_ Reduction and H_2_O Oxidation

Achieving high product yields in photocatalytic CO_2_ reduction without the need for sacrificial agents or additives is a significant challenge. As mentioned previously, the reduction of CO_2_ molecules to CO_2_^•−^ faces considerable difficulty due to its high potential. The introduction of sacrificial agents such as TEOA, MeOH, or Na_2_S can promote carbon dioxide reduction by providing protons and electrons to the system, thereby enhancing charge separation in the semiconductor. However, the use of such agents often leads to increased costs and reduced reliability. Additionally, the low solubility of carbon dioxide in water necessitates the exploration of reactions in organic media or the inclusion of basic additives to improve dissolution.

In this context, it becomes crucial to design catalysts that can efficiently reduce CO_2_ molecules using only water as a sacrificial agent. For this purpose, photocatalysts must possess excellent charge separation capabilities, enabling efficient migration toward CO_2_* molecules, along with appropriate band potentials that allow not only the oxidation of H_2_O but also the reduction of CO_2_ molecules, while preventing hydrogen evolution. The design of heterostructures has emerged as a potential approach to integrate these desired features. For example, Wong et al. developed a Z‐scheme utilizing α‐Fe_2_O_3_ and g‐C_3_N_4_ to enhance the selectivity of CO_2_ to CO without relying on sacrificial agents.^[^ ^161^ ^]^ The authors explained that this enhanced activity arises from a combination of improved electron‐hole separation and stronger CO_2_ adsorption on hematite. The effectiveness of heterostructures in enhancing CO_2_‐to‐CO reduction was further demonstrated by Li and colleagues.^[^ ^162^ ^]^ They revealed that the Local Surface Plasma Resonance of Au nanoparticles in the ZnO/Au/ g‐C_3_N_4_ system accelerates electron‐hole separation, resulting in high CO production without the need for additives. Additionally, it has been demonstrated that AgBr/BiOBr heterojunctions with surface oxygen vacancies, exhibiting excellent charge separation and strong redox ability, serve as outstanding photocatalysts for additive‐free CO_2_‐to‐CO reduction.^[^ ^163^ ^]^ In addition to oxides or oxyhalides, heterostructures of MOFs have been designed through hierarchical synthesis to form core‐shell MOF@MOF structures. These structures exhibit enhanced charge migration and large overpotential, enabling high selectivity for the production of HCOOH (146 µmol. g^−1^.h^−1^) without the need for sacrificial agents.^[^ ^164^ ^]^

Beyond utilizing H_2_O as an electron donor, a major aspiration in the scientific community is to develop an ideal catalyst capable of achieving artificial photosynthesis. This entails producing O_2_ from water simultaneously with the conversion of CO_2_ into other molecules. The main challenge lies in reconciling the complex CO_2_ activation with an appropriate band diagram capable of generating oxygen through H_2_O oxidation.^[^ ^165^ ^]^ The production of O_2_ is intricate due to the involvement of multielectron mechanisms. Lan et al. reported a CO/O_2_ production ratio of 2:1 using a Zn‐COF photocatalyst.^[^ ^166^ ^]^ The specific activity of this material is attributed to CO_2_ interaction with metal centers and H_2_O oxidation occurring at C = C and S atoms of the organic linker. In a subsequent study by the same research group, a pathway was proposed not only for CO production but also for H_2_O oxidation (Figure 15b). In this case, polyoxometalate clusters were incorporated into COF, leading to an O_2_ evolution mechanism involving H_2_O→OH→O→*OOH→O_2_.^[^ ^167^ ^]^ Furthermore, the “overall” CO_2_ reduction was demonstrated using BiOBr_x_Cl_1‐x_ catalysts. The authors suggested that both lattice and vacancy oxygens play a role in the mechanism, with lattice oxygen responsible for O_2_ generation and vacancy oxygen involved in oxygen lattice recovery and proton donation for CO production.^[^ ^168^ ^]^

### 5.3 CO_2_ Reaction Mechanisms

In recent years, there has been a growing interest in the conversion of CO_2_ to higher‐value C_2+_ products or C_1_ oxygenates, such as methanol or formic acid, as they possess greater economic value compared to commonly produced CO and CH_4_. However, achieving these higher compounds is challenging due to the complex kinetics involved. For instance, the production of CH_3_OH requires the generation and transfer of 6 e^−^ to the CO_2_ molecule.^[^ ^138^ ^]^ Despite these challenges, significant progress has been made in this field. Li and colleagues have reported the production of methanol and ethanol using a metal‐organic framework (UiO6‐NH_2_) supported with Cu single atoms.^[^ ^169^ ^]^ Additionally, Yang et al. demonstrated that polythiophene‐modified Bi_2_WO_6_ could enhance charge separation and generate methanol and ethanol through CO_2_ reduction.^[^ ^170^ ^]^ The presence of Co‐N_2_C single‐atom sites on g‐C_3_N_4_ has shown to be responsible for CH_3_OH production from CO_2_, as they can simultaneously capture electrons and adsorb carbon dioxide molecules.^[^ ^171^ ^]^

Producing C_2+_ compounds from CO_2_ reduction is particularly difficult due to the complexity of forming C‐C bonds. The key step to producing such compounds is the stabilization of two adsorbed intermediates to favor the coupling between these two species. For instance, Mo et al. synthesized dual single sites (P and Cu) supported on carbon nitride and verified the formation of the coupled intermediate *OC‐CHO, which is responsible for C_2_H_6_ production (616 µmol. g^−1^.h^−1^).^[^ ^172^ ^]^ Zhou et al. showed that Cu single atoms on Ti_0.91_O_2_ atomically thin single layers can produce hydrocarbons, such as propane. The authors explained that Cu‐Ti‐V_O_ (oxygen vacancies) can stabilize *CHOCO and *CH_2_OCOCO intermediates, facilitating the pathway for C‐C coupling.^[^ ^173^ ^]^ Furthermore, sulfur defects on the atomic layer of AgInP_2_S_6_ play a crucial role in accumulating charge on Ag sites and enhancing the adsorption of *CO molecules.^[^ ^174^ ^]^ This lowers the energy barrier for C‐C product formation, including ethene (Figure 15c). These findings highlight the significant advancements in the production of higher‐value oxygenates and C_2+_ compounds from CO_2_ reduction, despite the intricate nature of the reactions involved. Despite the intrinsic challenge of activating the CO_2_ molecule, its reduction via the direct use of green H_2_ in a solar‐to‐chemical approach is certainly the technology that holds the greatest potential among all the reactions discussed here. If successful, it can enable a path to decarbonization, leading to liquid fuels with no CO_2_ footprint, besides CO_2_ capture allowing in the best‐case scenario a route to revert global warming.

## 6 Conclusions, Challenges, and Perspectives

In summary, green hydrogen has significant potential as an energy carrier and key intermediate molecule for driving the chemical industry toward a more carbon‐neutral future. To achieve this goal, it is crucial to decouple the production of valuable chemicals from hydrogen derived from fossil fuels, which has a substantial carbon footprint. Creating a sustainable, decarbonized, and environmentally friendly chemical industry demands the exploration of viable alternatives. This perspective examines traditional fossil‐based processes and compares them to the potential of lab‐scale photocatalytic processes as a potential substitute for hydrogen derived from fossil fuels.

Currently, photocatalytic green hydrogen production faces challenges in competitiveness compared to electrolysis powered by renewable energy sources. However, a transformative shift occurs when we customize photocatalytic systems. These systems can be finely adjusted to utilize sunlight and renewable proton sources, leading to a direct solar‐to‐chemical approach. In recent examples, NH_3_, H_2_O_2_, CO_2_‐derived products, and chemicals produced through reduction reactions illustrate the potential of photocatalysis for creating valuable chemicals. While photocatalytic processes are not yet as efficient as commercial ones, advances in various related fields and interdisciplinary collaboration offer promising prospects for future improvements.

Today's technical challenges primarily revolve around the activity, engineering, and economic viability of photocatalysts. We believe that strategic adjustments to their structure‐activity relationships can further enhance photocatalysts. Recent strategies have shown significant improvements in photocatalyst efficiency, including: 1) defect/interface engineering, 2) cocatalyst integration, 3) heterojunctions, 4) atomic doping, and 5) active site modulation, among others. These approaches hold significant promise for increasing the efficiency of solar‐to‐chemical processes driven by photocatalysts. Given these challenges, we assert that solar‐to‐chemical processes are crucial for the chemical industry's transition to a low‐carbon economy.

Scaling up photoreactors to technological readiness levels 4‐ is also an important challenge that hinders the improvement of photocatalyst efficiency and broader acceptance in the field. Surprisingly, there is a lack of dedicated efforts focused on careful planning, simulation, and construction of pilot devices, and to a lesser extent, larger‐scale plants. However, it is clear that for making industrial‐scale photoreactors a reality, substantial research efforts in scaling up are essential. These efforts should aim to optimize factors such as photon utilization, mass transfer, heat management, and radiative profiles to ensure a reliable path toward large‐scale implementation.

Also, the search for renewable proton sources for solar to chemical synthesis is crucial in terms of process feasibility and circular economy. The use of diverse biomass waste materials, such as microplastics, crop residues, paper, and food or organic waste, is a very appealing solution for both utilizing protons and reducing environmental pollution. Moreover, it aligns with the circular economy by effectively closing the resource loop, especially when CO_2_ reduction reactions are involved.

All in all, the solar‐to‐chemical approach via photocatalysis offers a compelling route for collectively decarbonizing the chemical industry. In this context, strategic catalyst design and advancements in laboratory systems and photocatalytic devices will pave the way for establishing a sustainable and carbon‐neutral chemical industry. Therefore, the hydrogen economy is relevant in shaping the future of the energy landscape. It contributes to sustainability, facilitates the shift away from fossil fuels, reduces carbon emissions, and improves energy security. Nevertheless, realizing its complete potential requires us to prioritize addressing cost, infrastructure, and technological challenges.

## Conflict of Interest

The authors declare no conflict of interest.
